# Molecular Pharmacology and Novel Potential Therapeutic Applications of Fingolimod

**DOI:** 10.3389/fphar.2022.807639

**Published:** 2022-02-16

**Authors:** Safura Pournajaf, Leila Dargahi, Mohammad Javan, Mohammad Hossein Pourgholami

**Affiliations:** ^1^ Department of Physiology, Faculty of Medical Sciences, Tarbiat Modares University, Tehran, Iran; ^2^ Neuroscience Research Center, Shahid Beheshti University of Medical Sciences, Tehran, Iran

**Keywords:** fingolimod, immunomodulation, inflammation, apoptosis, autophagy

## Abstract

Fingolimod is a well-tolerated, highly effective disease-modifying therapy successfully utilized in the management of multiple sclerosis. The active metabolite, fingolimod-phosphate, acts on sphingosine-1-phosphate receptors (S1PRs) to bring about an array of pharmacological effects. While being initially recognized as a novel agent that can profoundly reduce T-cell numbers in circulation and the CNS, thereby suppressing inflammation and MS, there is now rapidly increasing knowledge on its previously unrecognized molecular and potential therapeutic effects in diverse pathological conditions. In addition to exerting inhibitory effects on sphingolipid pathway enzymes, fingolimod also inhibits histone deacetylases, transient receptor potential cation channel subfamily M member 7 (TRMP7), cytosolic phospholipase A2α (cPLA2α), reduces lysophosphatidic acid (LPA) plasma levels, and activates protein phosphatase 2A (PP2A). Furthermore, fingolimod induces apoptosis, autophagy, cell cycle arrest, epigenetic regulations, macrophages M1/M2 shift and enhances BDNF expression. According to recent evidence, fingolimod modulates a range of other molecular pathways deeply rooted in disease initiation or progression. Experimental reports have firmly associated the drug with potentially beneficial therapeutic effects in immunomodulatory diseases, CNS injuries, and diseases including Alzheimer’s disease (AD), Parkinson’s disease (PD), epilepsy, and even cancer. Attractive pharmacological effects, relative safety, favorable pharmacokinetics, and positive experimental data have collectively led to its testing in clinical trials. Based on the recent reports, fingolimod may soon find its way as an adjunct therapy in various disparate pathological conditions. This review summarizes the up-to-date knowledge about molecular pharmacology and potential therapeutic uses of fingolimod.

## Introduction

Fingolimod (FTY720, Gilenya) is a fungal metabolite derivate that was approved by the Food and Drug Administration (FDA) in September 2010 as the first orally administered disease-modifying drug for the treatment of relapsing-remitting multiple sclerosis (RRMS) (Chiba, 2020). Known as a sphingosine 1-phosphate (S1P) receptor modulator, fingolimod induces immunomodulation through lymphocyte sequestration (Brinkmann et al., 2004). However, more than two decades past fingolimod synthesis, it is thought that the mechanism (s) of action of fingolimod may be more than just lymphocytes confinement (Sica et al., 2019). This view partly stems from the studies reporting its effects on various diseases. While some recent reviews have focused on specific aspects of fingolimod actions and use, there is a lack of holistic review regarding piling up recent evidence in fingolimod pharmacology and potential applications. This review highlights the fingolimod history, development, pharmacological effects, and expanding potential therapeutic applications.

## Chemical Structure and Synthesis History

**FIGURE 1:**
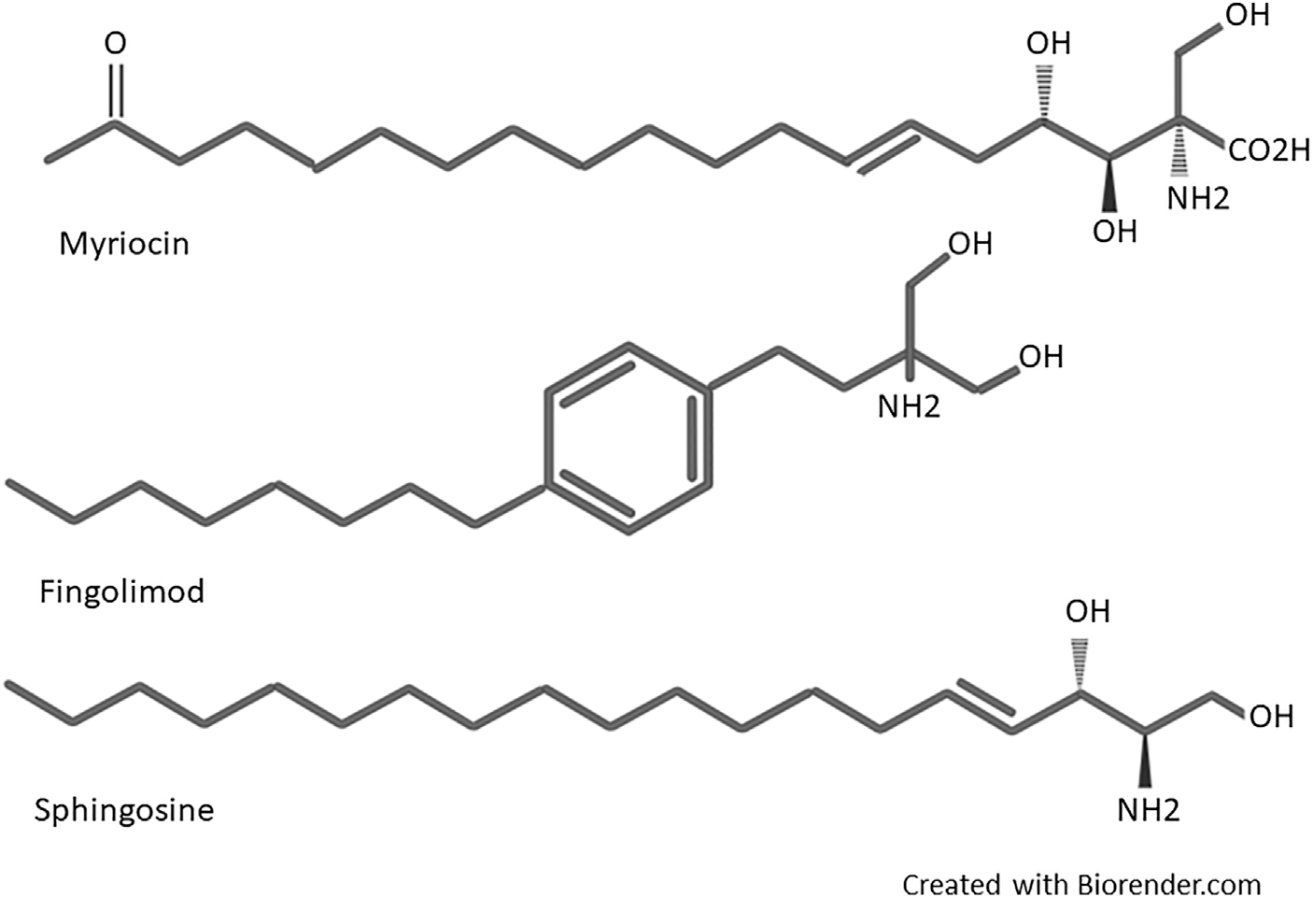
Structure of myriocin, fingolimod, and Sphingosine.

Fingolimod (2-amino-2[2-(4-octylphenyl) ethyl]-1, 3-propanediol) was first synthesized in 1995 by Adachi et al. from *Isaria sinclairii* metabolite named myriocin (ISP-1), a fungus used in Chinese traditional herbal medicine (Adachi et al., 1995). Structural simplification and modification of myriocin led to discovering several compounds with potent immunosuppressive activities more powerful than cyclosporine. During the modification process, the side chain functionalities and asymmetric centers of myriocin were removed, and a hydroxymethyl group instead of the carboxylic acid was substituted. In the next step, chimeric carbons elimination introduced much more simplified compounds. Then, the alkyl chains length were optimized. Finally, a phenyl ring inserted within the side chains introduced fingolimod (Chiba, 2020). Fingolimod was the most potent among these compounds and displayed noticeable immunosuppressive activity *in vivo* (Adachi et al., 1995). The structural components of fingolimod are different from conventional immunosuppressants, including an amino diol polar head group, a 1,4 di substituted phenyl ring, and a lipophilic alkyl tail (Brinkmann et al., 2010). A more detailed structural description of fingolimod is available in Marciniak’s work (Marciniak et al., 2018). As it can be seen in Figure 1, a highly close structural resemblance exists between fingolimod and Sphingosine, a member of the sphingolipid family and metabolite of sphingomyelin (a major component of the cell membrane), which led to the idea of fingolimod action on S1P receptors as the principal mode of action (Mandala et al., 2002).

## Pharmacokinetics

Following oral administration, slow and food-independent absorption of fingolimod is achieved with around 93% oral bioavailability and maximal plasma concentration reached after 12–16 h. In the body, fingolimod is metabolized via three main pathways. The first is rapid and reversible phosphorylation by sphingosine kinases (SPHKs) to fingolimod-phosphate (fingolimod-P). Lipid phosphate phosphohydrolases (LPP1a and LPP3) dephosphorylate fingolimod-P to fingolimod. Specific sphingosine 1-phosphate phosphatase (SPP1) is also involved in intracellular dephosphorylation of fingolimod-P to a lower degree. The second is hydroxylation/oxidation by cytochrome P450 (CYP) 4F2 to inactive metabolites, eliminated by the renal system (81%). In the third pathway, inactive non-polar ceramides are produced by the (dihydro) ceramide synthase and ceramidase for the reverse reaction. Generally, fingolimod-related components detected in the blood are fingolimod (23.3%) and fingolimod-P (10.3%), followed by inactive metabolites butanoic acid (8.3%), ceramide metabolites M29 (8.9%), and M30 (7.3%). In the urine, butanoic acid is the major recovered metabolite (36%) of the total dose administered (David et al., 2012). The parent drug fingolimod highly distributes in red blood cells (86%), while in its phosphorylated form, fingolimod-P has lower uptake into red blood cells (17%). The distribution of fingolimod in body tissues is widespread, with a distribution volume of about 1,200±260 L. Fingolimod has >99.7% plasma protein binding (mainly albumin) (David et al., 2012). While there are no reports showing fingolimod binding to ApoM/HDL, S1P ApoM/HDL binding seems essential for exerting its physiological effects (Christoffersen and Nielsen, 2013). Slow blood clearance results in an average terminal half-life of 6–9 days after repetitive administration. One-to-two months after once-daily drug intake, steady-state blood accumulation will be nearly 10-fold higher than the concentrations achieved following the initial dose (David et al., 2012). Having the same elimination profile, fingolimod and fingolimod-P levels stay nearly equal in the blood (Volpi et al., 2019).

## Safety, Adverse Effects, and Contraindications

Fingolimod treatment is usually well tolerated. However, by virtue of acting on the various S1P receptors, fingolimod induces numerous biological effects, including endothelial cell-cell adhesion, angiogenesis, vascular integrity, and cardiovascular function. The most common side effects observed in clinical studies in multiple sclerosis (MS) are reported to be lymphopenia, influenza, infections, nasopharyngitis, fatigue, back pain, diarrhea, bronchitis, dyspnea, nausea, and abnormal liver function tests (Kappos et al., 2006). Bradycardia with a possible atrioventricular blockade is a transient side effect observed following the first dose of fingolimod. Thus, pulse rate and blood pressure monitoring for at least 6 hours following the first application is needed (Széplaki and Merkely, 2012). In addition, during the first 3–4 months of fingolimod treatment, macular edema with or without visual symptoms has been reported (Yeh and Weinstock-Guttman, 2011). Naturally reducing peripheral lymphocyte count, fingolimod use may increase the risk of infections. So, patients must be warned about the signs of infection during and until 2 months post-fingolimod treatment (Yeh and Weinstock-Guttman, 2011; Fazekas et al., 2012). Fingolimod is a pregnancy risk category C drug. Congenital abnormalities have also been reported as fingolimod adverse effects in animal studies. Hence, in Europe, the drug is contraindicated in pregnancy, and its discontinuation is highly recommended at least 2 months before inception (Gilenya Contraindicated in Pregnant Women in EU, 2019; Mendibe Bilbao et al., 2019). Peripheral-vascular adverse effects like purplish blotches, itching, and edema on the distal phalanges are also among the rare adverse effects of the drug (Russo et al., 2015).

## Mechanisms of Action

**FIGURE 2:**
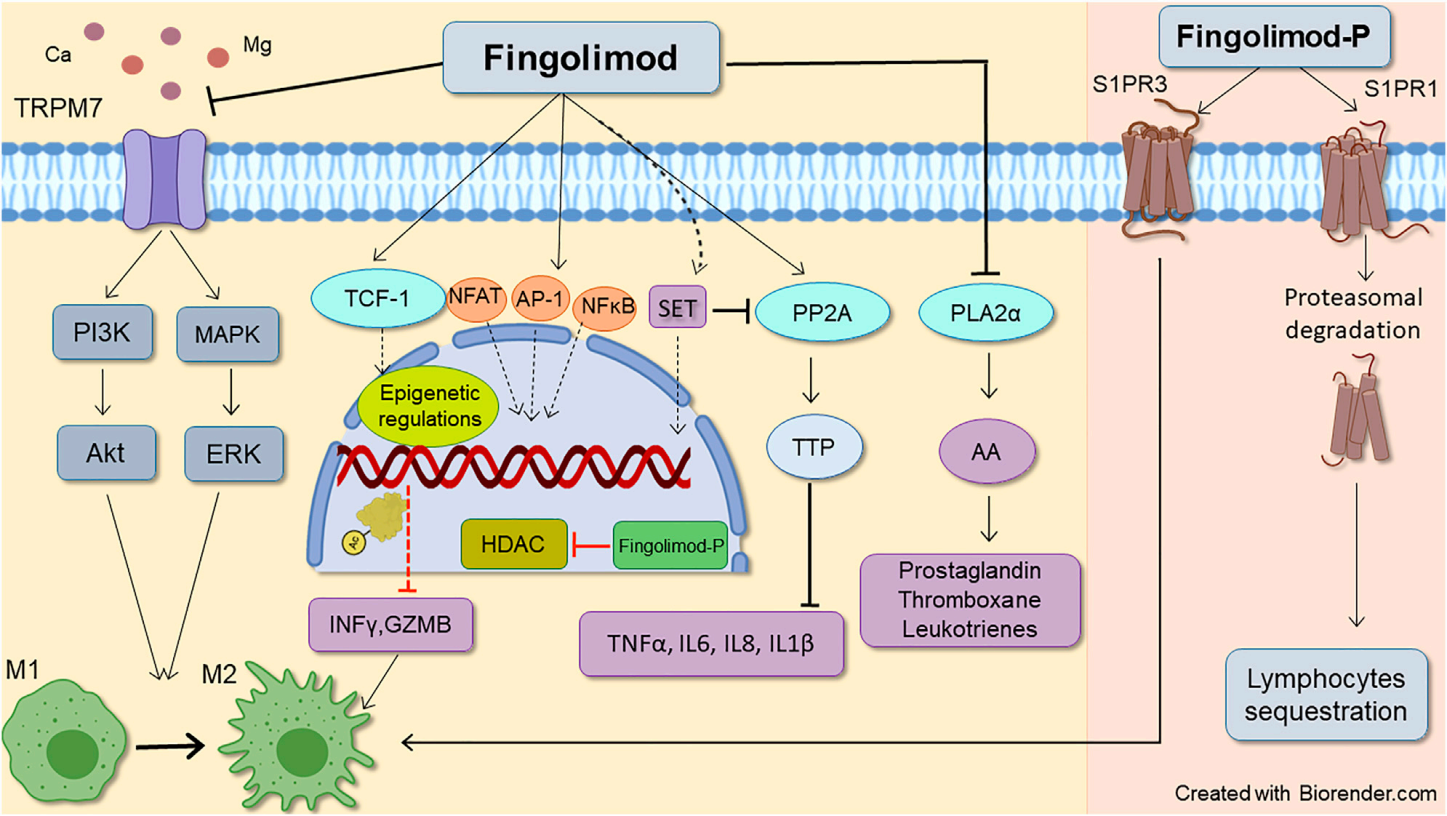
Schematic representative of fingolimod anti-inflammatory mode of action. Fingolimod main immunomodulatory action occurs through downregulating S1P receptors1, leading to lymphocytes sequestration. Its action on S1P receptors3 also favors lymphocytes M2 anti-inflammatory phenotype which is mediated by STAT3 phosphorylation. Besides, non-S1P related actions of fingolimod also can reduce inflammatory response by decreasing inflammatory molecules, T-cell inhibition, and shifting microglia and macrophages toward M2 phenotype. Enhancing histone acetylation by epigenetic regulation, phospholipase A2α inhibition, and activation of PL2A (through inhibition of SET expression) by fingolimod reduces inflammatory cytokines such as TNFα, IL6, IL8, and IL-1β. The drug also inhibits PLA2α (which contributes to AA release and subsequent prostaglandins production), and TRPM7 chanzyme (which can induce both pro and anti-inflammatory phenotypes in macrophages). AA, Acid arachidonic; AP-1, Activator protein 1; Fingolimod-P, Fingolimod phosphate; GZMB, granzyme B; HDACi, Histone deacetylate inhibition, IL (6, 8), Interleukin (6, 8); INFϒ, interferon-gamma; M1, Pro-inflammatory macrophage/microglia; M2, Anti-inflammatory macrophage/microglia NFAT1, Nuclear factor of activated T-cells 1; NFκB, Nuclear factor-kappa B; PLA2α, Phospholipase 2α; PP2A, Protein phosphatase 2A; TCF-1, T cell factor 1; TNFα, Tumor necrosis factor alpha; TTP, Tristetraprolin.

Sphingosine kinase 2 (SPHK2) and with lesser efficacy (30 folds lower), sphingosine kinase1 (SPHK1) can phosphorylate fingolimod to Fingolimod-P (Billich et al., 2003; Paugh et al., 2003). Fingolimod-P binds with high affinity (EC_50_ ∼ 0.3–3 nM) to G-protein coupled receptors (GPCRs) S1PR (1, 3, 4, and 5), except S1P receptor 2, which shows a very low affinity with EC_50_ values more than 10,000 nM (Brinkmann et al., 2002; Mandala et al., 2002; Albert et al., 2005). Both SPHKs phosphorylate sphingosine to S1P within the cell. S1P is then exported outside the cell with the aid of ABC transporters and spinster 2 transporter to act on all S1PR1-5 (Nagahashi et al., 2014). S1P is a pivotal molecule in intracellular signaling (Lee et al., 1998; Maceyka et al., 2005), and based on the location of production, has distinct functions (Kumar et al., 2017). S1P phosphatase, located in the endoplasmic reticulum (Hla et al., 2008), and S1P lyase, found in both nucleus and endoplasmic reticulum (Ebenezer et al., 2021), maintain the intracellular concentration of S1P (Hla et al., 2008). The first dephosphorylates S1P to sphingosine, and the latter irreversibly degrades S1P to ethanolamine phosphate and hexadecenal (Spiegel, 2000). While interstitial fluid levels of S1P are low, S1P is highly augmented in blood and lymph in the sub-micromolar range (∼1 µM), generating an intense S1P gradient (Hla et al., 2008). Mediated also by SPHKs, and S1P transporters (Tsai and Han, 2016), this S1P gradient, along with surface residence of S1PR1 on immune cells, sets as a cue for regulating immune cells such as lymphocytes and hematopoietic progenitor cells egress process (Obinata and Hla, 2019); thus, disruption of this principal gradient can induce lymphopenia by interfering in lymphocytes trafficking (Schwab et al., 2005). Affinity and potency of S1P and fingolimod-P for S1PR isoforms differ (fingolimod-P has high potency for S1PRs and has higher efficacy than S1P). Therefore, it promotes distinct responses in the target cells (Huwiler and Zangemeister-Wittke, 2018). Figure 2 depicts the primary mechanism through which fingolimod modulates inflammatory responses (further discussed in the following sections).

### Sphingosine 1 Phosphate Receptors

#### S1PR1

The affinity of fingolimod-P for the S1P receptor1 (S1PR1) is high (Brinkmann et al., 2002), which is the first identified and the most studied S1P receptor (Roggeri et al., 2020). It is the only S1PR that couples exclusively to Gαi/o (Lee et al., 1998). Binding to Gαi/o, it activates numerous signaling pathways including phosphatidylinositol-3-kinase (PI3K)/Akt, PI3K/Rac, signal transducer and activator of transcription (STAT3), and phospholipase C (PLC), which are involved in cell survival, proliferation, and migration (Wang et al., 2019). The most prominent functions of S1PR1 occur on lymphocytes, natural killer cells, dendritic cells, macrophages, neutrophils, hematopoietic progenitors, mast cells, and osteoclasts (Matloubian et al., 2004; Kihara et al., 2014). Detecting high levels of S1P in the blood and lymph, S1PR1 initiates egress of the lymphocytes from the lymphoid organs into the blood via the Gαi PI3K pathway and the small GTPase Rac activation (Cyster and Schwab, 2012). Down-regulation or desensitization of S1PR1s activates lymphocyte migration from the blood into the tissues. S1PR1 signaling regulates downstream molecules of the pro-survival PI3K/Akt proteins, necessary for apoptosis prevention (Schwab and Cyster, 2007). Binding S1P to S1PR1 activates the Rho family of small GTPases and downstream assembly of adherens junction and tight junction, and focal adhesion formation (Wang and Dudek, 2009). In humans, S1PR1 affects cardiovascular function and is the primary driver of bradycardia. At the very first hours of use, fingolimod acts as S1PR1 agonist and activates G-protein-coupled inwardly-rectifying potassium (GIRK) channels on atrial myocytes and endothelial cells, leading to bradycardia, eNOS activation, and ultimately, blood pressure decrease (Ochi et al., 2006; Fryer et al., 2012). However, this is transient as functional antagonistic activity of fingolimod on S1PR1 appears. The dominant and first known effect of fingolimod is immunomodulation through lymphocyte sequestration. Unlike conventional immunomodulatory drugs that act on calcineurin, fingolimod does not affect the proliferation and activation of lymphocytes. Instead, it induces a marked decrease of circulating lymphocytes (Chiba et al., 1998). As a pharmacological agent, the mechanism through which this happens is quite exciting and perhaps seldom seen. Upon fingolimod-P or S1P binding to the S1PRs, all receptors are internalized, dissociated in endosomes, and then recycled back to the cell membrane, except S1PR1, which is permanently downregulated by fingolimod-P (S1P lyase catabolizes the S1P, but not the fingolimod-P). Then through consistent binding, fingolimod-P induces its functional antagonistic effects leading to lymphocytes traffic perturbation (Schwab et al., 2005; Brinkmann, 2007). With the high affinity for the S1PR1, fingolimod-P causes irreversible receptor downregulation. Subsequently, desensitization to the serum S1P occurs and eventually leads to lymphocyte immobilization in the lymphoid organs and reduces their infiltration into the central nervous system (Matloubian et al., 2004). This persistent internalization is brought about through strong polyubiquitination by fingolimod-P and consequent degradation of S1PR1, evoking a massive decline in the S1PR1 level (Oo et al., 2007). In contrast, S1P causes c-terminal tail phosphorylation of S1PR1, inducing the agonistic response of S1PR1, leading to temporary internalization via clathrin-mediated endocytosis (Liu et al., 1999). S1PRs1 are highly expressed on the endothelial cells, supporting vascular development and endothelial barrier integrity augmentation (Proia and Hla, 2015). By acting on S1PR1, fingolimod maintains vascular integrity through enhancing adherens junction assembly and endothelial barrier function (Brinkmann et al., 2004). Upon treatment of endothelial cells with fingolimod-P, phosphorylated extracellular signal-activated kinase (pERK) and pAkt lead to cell survival. Furthermore, vascular permeability induced by vascular endothelial growth factor (VEGF) is blocked by fingolimod (Sanchez et al., 2003). As a consequence of allergy, inflammation, or cancer, vascular permeability increases, hence barrier protective properties of S1P signaling are of therapeutic interest (Huwiler and Zangemeister-Wittke, 2018). In experimental autoimmune encephalomyelitis (EAE), fingolimod decreased the matrix metalloproteinase (MMP) gene-9 and increased its counter regulator, tissue inhibitor of metalloproteinase (TIMP-1). Resulting in preserving blood-brain-barrier (BBB) integrity (Foster et al., 2009). In a murine model of gut ischemia/reperfusion (I/R), fingolimod protected against the inflammatory cascade by protecting vascular barrier integrity (Potì et al., 2020). In an animal model of septic shock, fingolimod diminished levels of Evans blue leakage from the blood into the liver and lung, decreased hematocrit values, and lowered plasma levels of VEGF-A (Hemdan et al., 2016). However, while S1PR1s are known to strengthen adherens junctions between endothelial cells (Gaengel et al., 2012) and maintain vascular barrier integrity, involvement of other receptor subtypes are also suggested (Potì et al., 2020). It has been shown that fingolimod improves the pulmonary endothelial cell barrier in a way independent of S1PR1 ligation (Dudek et al., 2007). Preservative effects of fingolimod on endothelial integrity seem to be dose-dependent. While low concentrations tend to be barrier augmentative, higher concentrations may induce irreversible barrier breakdown accompanied by induction of apoptosis (Müller et al., 2011). Other preclinical studies have also reported induction of vascular leakage at higher concentrations (Oo et al., 2011; Wang et al., 2014). These contradictory effects of fingolimod suggest that agonistic activity of S1PR1 augments endothelial barrier integrity while antagonist activity of fingolimod (which occurs after prolonged exposure) induces barrier disruption and increases vascular leakage (Huwiler and Zangemeister-Wittke, 2018). Moreover, fingolimod has been shown to inhibit angiogenesis under both *in vitro* (Ho et al., 2005) and *in vivo* (Schmid et al., 2005) conditions leading to antitumor effects (Chua et al., 2005; Ho et al., 2005; Schmid et al., 2005; LaMontagne et al., 2006). As S1P seems to play a dominant role in angiogenesis, fingolimod as its regulator can thus be regarded as an antiangiogenic. It has been shown that fingolimod impairs angiogenesis in mechanical force-induced abnormal scars (Aoki et al., 2020) and inhibits tumor angiogenesis via the S1PR1 (Schmid et al., 2007). Interestingly, depending on the setting and context, there are reports that fingolimod may also promote angiogenesis, where the phenomenon leads to a therapeutic effect. One such example is the photothrombotic model of mice brain ischemia, where fingolimod treatment enhanced angiogenesis by modulation of the microglial anti-inflammatory phenotype (M2) polarization *in vivo* and *in vitro* (Shang et al., 2020). In line with this, fingolimod promoted angiogenesis in mice after stroke (Zou et al., 2020). Following fingolimod use, S1PR1 dependent endothelial NOS (eNOS) activation and eNOS release lead to short-term vasodilation and subsequent blood pressure drop. This is why some patients experience a transient reduction in blood pressure when starting fingolimod therapy (Camm et al., 2014). Another way of regulating S1PR1 by fingolimod is through STAT1. It has recently been shown that fingolimod can suppress lipopolysaccharide (LPS) induced STAT1 activity (Hu et al., 2021), and STAT1 can transcriptionally stimulate S1PR1 by binding its promoter region (Xin et al., 2020). Additionally, activation of S1PR1 is involved in opioid-induced-hyperalgesia and fingolimod, by antagonistic effects on S1PR1, attenuates the development of morphine-induced persistent neuropathic pain in rats (Doyle et al., 2020).

#### S1PR3

S1P receptor3 (S1PR3) recruits multiple G-proteins, including Gαi/o, Gα12/13, and Gαq, with the most frequent action on the latter (Chun et al., 2010). The coupling of Gαq to S1PR3 generates inositol trisphosphate and diacylglycerol. Then calcium mobilization and activation of PKC, respectively, occur (Kihara et al., 2014). S1PR3 also contributes to angiogenesis, although less strongly than S1PR1 (Lee et al., 1999). It has been shown that S1PR3 depletion inhibits neurite retraction, suggesting a role of S1PR3 in nerve regeneration (Anastasiadou and Knöll, 2016; Quarta et al., 2017). Bradycardia induction has been attributed to S1P3 modulation. However, it is a species-specific function only seen in mice and rats (Sanna et al., 2016). In rats, bradycardia and hypertension induced by fingolimod are subtype-specific, attributed to S1PR1 and S1PR3, respectively, (Forrest et al., 2004; Fryer et al., 2012). Phagocytosis, polarization to M1 Phenotype, and reactive oxygen species (ROS) production in macrophages occur through S1PR3 (Hou et al., 2017; Gaire et al., 2018; Bryan et al., 2020). S1PR3 has been recognized as the receptor responsible for fingolimod-induced arterial vasodilation (Tölle et al., 2005). S1PR3 in macrophages can be considered an inflammatory receptor (Weigert et al., 2019). Fingolimod treatment interferes with S1PR3 signaling by opposing Gαq, leading to reduced ROS production and phagocytosis and M2 polarization of macrophages (Bryan et al., 2020). Amelioration of neuroinflammatory response occurs through fingolimod’s ability to polarize microglia toward the M2 anti-inflammatory phenotype (Shang et al., 2020). This microglial polarization toward the M2 phenotype is mediated by the STAT3 pathway (Qin et al., 2017). Fingolimod-P’s protective role in astrocytes against oxygen-glucose deprivation-induced neuroinflammation is also S1PR3-dependent, done through TLR2/4-PI3K-NFκB signaling pathway inhibition (Dong et al., 2018). It has been suggested that, like S1PR1, S1PR3 is also has a role in cancer metastasis (Calis et al., 2017). S1PR3 upregulation occurs in different cancers like breast cancer and brain tumor metastasis (Filipenko et al., 2016; Gril et al., 2018). So, it is intriguing that fingolimod by modulating S1PR3 and S1PR1s may provide anticancer effects.

#### S1PR4

Fingolimod binds to S1P receptor4 (S1PR4) that has a restricted distribution in the body (Stepanovska et al., 2020) and is primarily expressed in lymphocytes and hematopoietic tissues. Specifically abundant in immune cells, S1P/S1PR4 axis plays a significant role in immunity (Olesch et al., 2017). Recent studies emphasize the S1PR4 signaling role in activating immune cells differentiation and trafficking (Olesch et al., 2017). It acts on Gαi and Gα12/13, which induces mitogen-activated protein kinase (MAPK) activity, and activates RhoA/ROCK. Activating RhoA/ROCK affects actin dynamics and regulates trafficking of other receptors as a typical feature of S1PR4 biological action in immune cells (Olesch et al., 2017). In addition to S1PR1, S1PR4 also plays a crucial role in mediating the S1P migratory effect on satellite cells (Calise et al., 2012). It has a role in neutrophil traffic (Maceyka and Spiegel, 2014) as S1PR4 knockout in mice and zebrafish demonstrate reduced amounts of circulating neutrophils (Pankratz et al., 2016). S1PR4 mediates immunosuppressive effects of S1P through inhibition of T cell proliferation and modulation of cytokines (Wang et al., 2005). In addition to S1PR1, S1PR4 is also involved in the migratory response of migratory T cells toward S1P (Matsuyuki et al., 2006). S1PR4 signaling regulates the function of dendritic cells and Th17 T-cell differentiation (Schulze et al., 2011). It has recently been shown that S1PR4 deficiency affects the migration and positioning of activated peritoneal B cells to secondary lymphoid organs resulting in a significant drop in numbers of splenic innate response activator B cells after LPS-induced peritonitis (Riese et al., 2021). S1PR4 physiological roles are not yet fully elucidated. Hence, fingolimod effects through S1PR4 signaling are unclear so far (Stepanovska et al., 2020).

#### S1PR5

Fingolimod binds with high affinity to S1P5 receptor5 (S1PR5), which has limited expression in the CNS (Ishii et al., 2004). After coupling with multiple G-proteins, the S1PR5 binds primarily to Gα12/13. Functional consequences of S1PR5 modulation are associated with activation of signaling pathway molecules such as ERK1/2 (Jaillard et al., 2005). S1PR5 is dominantly expressed in oligodendrocytes and NK cells (O'Sullivan and Dev, 2017) and increases the survival of mature oligodendrocytes (Jaillard et al., 2005). Besides inhibition of oligodendrocyte precursor cell migration (Novgorodov et al., 2007), through S1PR1s and S1PR5s regulation, fingolimod also regulates survival, differentiation, and the extension of the cellular process in oligodendrocyte progenitors (Jaillard et al., 2005; Miron et al., 2008). It has been shown that fingolimod can decrease the expression of S1PRs, specially S1PR5, thus inhibiting the proliferation of multiple myeloma cells (Fu et al., 2017). Expression of S1PR5 on brain endothelial cells enhances the integrity of the brain endothelial barrier (van Doorn et al., 2012). S1PR5 also promotes lymphocytes and NK cell trafficking and their guidance to the inflammation sites, like S1PR1 and S1PR3 (Walzer et al., 2007).

### Sphingolipid Pathway Enzymes

Although fingolimod exerts much of its therapeutic effects through modulation of S1PRs, other sites of action have also been suggested (Ntranos et al., 2014; Mazzola et al., 2015). Specific protein-binding, intracellular signaling pathways activation, and epigenetic transcriptions modulation are among these receptor-independent mechanisms of fingolimod (Hait et al., 2014).

#### SPHKs

Fingolimod, but not fingolimod-P, is a competitive SPHK1 inhibitor (to sphingosine) and induces its ubiquitin-proteasomal degradation in HPASMC, MCF-7, and LNCaP-AI cells (Tonelli et al., 2010). Moreover, fingolimod can inhibit SPHK2 in neuroblastoma cells (Li et al., 2013). These two enzymes have different developmental and tissue distribution and play opposing roles in cell survival and apoptosis (Maceyka et al., 2005). Predominantly cytosolic, SPHK1 is anti-apoptotic and pro-survival, while SPHK2 is mainly localized in the plasma membrane, and the nucleus is pro-apoptotic (Kumar et al., 2017). Since SPHK1 activation is associated with cancer development with poor prognosis (Pyne and Pyne, 2010), the ability of fingolimod in inhibiting SPHK1 is quite intriguing and needs further investigations. However, fingolimod IC_50_ for SPHK1 is 50 µM concentration with no clinical relevance. That encourages the search for drugs with higher potency for SPHK1 inhibition.

#### S1P Lyase

Fingolimod can inhibit S1P lyase activity (Bandhuvula et al., 2005) that induces S1P irreversible degradation, so its pharmacological inhibition by fingolimod can change the S1P chemotactic gradient that plays in thymocytes egression (Schwab et al., 2005). Alongside affecting S1P signaling, S1P lyase affects different biological functions involved in cell survival, migration, inflammation, and oncogenesis. S1P lyase deficiency in gut epithelial cells has been associated with colitis and the development of colitis-associated cancer (Kumar et al., 2017). Hence, its inhibition by fingolimod has to be considered in cancer therapy.

#### Ceramidase Synthase

Modulation of ceramide synthesis is another action of fingolimod (Lahiri et al., 2009). Fingolimod inhibits ceramide synthase 2 in a competitive behavior toward dihydrosphingosine (Berdyshev et al., 2009) and is noncompetitive toward acyl-CoA. It also modulates the intracellular balance of signaling sphingolipids. Fingolimod inhibits ceramide synthesis at high (500 nM–5 µM) but not low (<200 nM) Sphinganine concentrations, which seems to be acyl-CoA chain length-dependent (Lahiri et al., 2009). It also has been shown that fingolimod reduces the light-induced retinal ceramide increase by *de novo* ceramide biosynthetic inhibition (Chen et al., 2013).

#### Acid Sphingomyelinase

Through proteolytic degradation, fingolimod inhibits enzyme acid sphingomyelinase (A-SMase) as a catalyzer of hydrolysis of sphingomyelin to ceramide. However, this effect seems indirect and cumulatively increased (Dawson and Qin, 2011; Henry et al., 2013). By reducing A-SMase activity, fingolimod decreases the production of monocytes-derived micro-vesicles and IL-1β in patients with MS (Amoruso et al., 2018). Extracellular vesicles participate in BBB dysfunction, and the accelerated level of extracellular vesicles in biological fluids of MS patients contributes to MS pathogenesis (Dolcetti et al., 2020). This mode of action of fingolimod deserves further investigation.

### Epigenetic Regulation: Histone Deacetylase Inhibition

Epigenetic regulation is also involved in the receptor-independent functions of fingolimod. Fingolimod acts as a histone deacetylases inhibitor (HDACi) (Hashemian et al., 2019; Ji et al., 2019; Rohrbach et al., 2019). After phosphorylation in the nucleus, fingolimod binds and inhibits class 1 histone deacetylases (HDACs), enhancing specific acetylation (Ji et al., 2019) and rescuing memory deficits independent of its immunosuppressive actions (Hait et al., 2014). Clinically relevant oral doses of fingolimod (1 mg/kg) suppressed development, progression, and aggressiveness of spontaneous breast tumors in MMTV-PyMT transgenic mice and reduced HDAC activity that reactivated estrogen receptor-α (ERα) expression (Hait et al., 2015). Preliminary evidence shows that besides H1 acetylation enhancement by fingolimod, H3 histone acetylation is also increased. Fingolimod increased H3 acetylation at brain-derived neurotrophic factor (BDNF) promoter in OLN-93 cell line and increased BDNF expression in oligodendroglial cells (Segura-Ulate et al., 2017). Antitumor activity in medulloblastoma cells treated by 7.5 or 10 µM concentrations of fingolimod attributed to the induced H3 acetylation (Perla et al., 2020). It has also been shown that after myocardial infarction, HDAC inhibition induces M2 macrophages and increases non-inflammatory cytokines alongside angiogenesis (Kimbrough et al., 2018). HDAC inhibition by fingolimod also causes a reduction in activated T cells, upregulation in antiepileptogenic effect, neurotrophic factor generation enhancement, and memory deficit rescue (Hait et al., 2014; Leo et al., 2017; Segura-Ulate et al., 2017; Baer et al., 2018). Fingolimod induces expression of T cell factor 1 (TCF-1), which then binds to the promoter/enhancer regions and causes inhibition of some inflammatory genes like interferon-gamma (IFN-ϒ) and granzyme B (GZMB) (Mazzola et al., 2015). Besides, fingolimod induces parts of its neuroprotective effects by enhancing neurotrophic factors. Fingolimod enhances BDNF expression *in vitro* in different cells, including epidermal neural crest stem cells (EPI-NCSCs) (Pournajaf et al., 2020), primary mouse cortical neurons (Doi et al., 2013), microglia (Noda et al., 2013), Schwann cells (Heinen et al., 2015), and oligodendrocytes (Segura-Ulate et al., 2017). Fingolimod-induced enhancement of BDNF has also been shown. In a mouse model of Rett syndrome, fingolimod counteracted NMDA-induced neuronal death in a BDNF-dependent manner (Deogracias et al., 2012). Fingolimod reduced synucleinopathy in mouse models of Parkinson’s disease and improved behavior by increasing BDNF levels (Vidal-Martínez et al., 2016; Vidal-Martinez et al., 2019). In an animal model of MS, fingolimod protected brain tissue from atrophy attributed to the promotion in the BDNF (Smith et al., 2018). In patients with MS, treatment with fingolimod significantly increased BDNF secretion from T cells, suggesting that neuroprotective effects of fingolimod therapy are through BDNF enhancement (Golan et al., 2019). Fingolimod regulates dendritic architecture and morphology of healthy mature primary hippocampal neurons alongside BDNF dependent enhancement in c-Fos and pERK1/2 proteins (Patnaik et al., 2020). We have recently shown that BDNF enhancement by fingolimod correlates with upregulation in oligodendrocyte mRNA levels in the EPI-NCSCs (Pournajaf et al., 2020). BDNF activates tropomyosin-related kinase B (TrkB), the MAPK/ERK1/2 signaling, and eventually causes oligodendrocyte differentiation and myelination (Fletcher et al., 2018). This myelination enhancement can partly explain the neuroprotective effects of fingolimod. Histone deacetylase inhibitory ability of fingolimod is involved in BDNF enhancement. The BDNF gene reacts to different epigenetic factors, especially HDAC inhibitors (Bagheri et al., 2019). Recently, it has been reported that HDAC inhibition increases neurotrophic (especially BDNF) expression after traumatic brain injury (TBI) (Sada et al., 2020). Segura-Ulate and others also found that fingolimod increases histone H3 acetylation in OLN-93. With the aid of chromatin immunoprecipitation assays, they found acetylated histone 3 enhancement at BDNF promoter-1 after fingolimod exposure, suggesting that fingolimod-associated histone deacetylase inhibition stimulates BDNF expression in oligodendroglia cells (Segura-Ulate et al., 2017). However, in an MTPT mouse model of Parkinson’s disease, fingolimod did not increase BDNF sustainably (Komnig et al., 2018). Moreover, in a recent trial evaluating fingolimod in patients with Rett syndrome (FINGORETT study), 12-month treatment with the drug did not lead to BDNF enhancement in children with Rett syndrome (Naegelin et al., 2021). Therefore, further trials under different clinical settings are required to determine whether or not fingolimod treatment leads to BDNF enhancement.

Besides decreasing S1PR1 expression in macrophages that cause phenotypic transformation of macrophages from a pro-inflammatory (M1) to M2 anti-inflammatory phenotype (Hughes et al., 2008), fingolimod facilitates M1 to M2 shifts of macrophages and microglia (Sun et al., 2018; Ji et al., 2019), enhances their phagocytic function, and modulates their proliferation, morphology, and cytokine release via suppressing HDAC1-Mediated Krüppel-like factor 4 (KLF4) deacetylation (Ji et al., 2019). However, inhibition of the transient receptor potential cation channel, subfamily M, member 7 (TRPM7) by fingolimod prevents polarization of macrophages towards the anti-inflammatory M2 phenotype (Schilling et al., 2014). Suppressing autophagy via the STAT1 pathway is considered another mechanism of fingolimod-mediated microglial transform to an anti-inflammatory phenotype (Hu et al., 2021). Along with changing absolute lymphocyte numbers, fingolimod selectively changes peripheral blood lymphocyte subsets. Indicating that although the number of peripheral memory lymphocytes is reduced by fingolimod, significant parts of the immunological memory are preserved (Hjorth et al., 2020).

Another effect of fingolimod is modulating T cell phenotype and regulatory T cell plasticity. In patients with RRMS, 0.5 mg/kg of fingolimod induces an exhausted-like phenotype, characterized by the inhibition of IL-17 and IFNγ expression, augmentation of IL-10 and TGFβ, and increased expression of exhaustion markers such as programmed cell death-1 (PD-1) and T-cell immunoglobulin and mucin domain-containing protein 3 (Tim-3) (Dominguez-Villar et al., 2019). Suppression of T cell activation by fingolimod occurs through inhibition of distal T cell receptor signaling. As shown in Figure 2, aberrant nuclear translocation and activation of nuclear factor of activated T-cells 1 (NFAT1), activator protein 1 (AP-1), and nuclear factor-kappa B (NFκB) by fingolimod enhances histone H3 lysine 9 acetylation (H3K9Ac), leading to T cell inhibition and immunomodulation (Baer et al., 2018).

### The Chanzyme TRPM7 Inhibition

TRPM7 is an ion channel and protein kinase that, by regulating Ca^2+^ and Mg^2+^, mediates several physiological and pathological processes like cell proliferation, survival, migration, and apoptosis (Abumaria et al., 2019). Mounting evidence implies that TRPM7 chanzyme is critical in several aspects of cancer (Yee, 2017). Fingolimod is a potent inhibitor of TRPM7 (IC_50_ = 0.72 µM) that contributes to the drug’s anti-proliferative and anti-migratory effects (Qin et al., 2013). TRPM7 is abundant in macrophages, and its activation is essential for the polarization of macrophages into anti-inflammatory M2 phenotype. As shown in Figure 2, pharmacological inhibition of TRPM7 by fingolimod hinders macrophage M1/M2 shift (Schilling et al., 2014). It has also been suggested that loss of TRPM7-mediated Ca^2+^ influx in response to LPS after TRPM7 inhibition is involved in macrophage inactivation (Schappe et al., 2018). Moreover, TRPM7 inhibition decreases PI3K and ERK1/2 phosphorylation (Fang et al., 2013), required for macrophages to shift towards the M2 phenotype (Zhang et al., 2011). Inhibiting TRPM7 channel activity by fingolimod causes IL-2 sensitization, leading to upregulation of Foxp3 in thymocytes and the development of T regulatory (Treg) cells (Mendu et al., 2020). However, recently Li et al. showed the opposite role of TRPM7 in macrophage polarization, suggesting that M1 macrophages highly express TRPM7, and its blockade could drive macrophages to M2 polarization. The proposed mechanism for this regulation of macrophage phenotype is thought to involve inhibition of STAT1 phosphorylation and promotion of STAT6 phosphorylation after TRPM7 inhibition (Li L. et al., 2020). It has been shown that pathological triggers such as ischemic, hypoxic, and traumatic injuries can over-activate TRPM7, leading to neuronal death and toxicity (Abumaria et al., 2019). Fingolimod inhibitory effects on TRPM7 can be investigated for new therapeutic applications in diseases affected by these triggers.

### Cytosolic Phospholipase A2α Inhibition

Part of fingolimod-induced changes occurs by inhibition of cytosolic phospholipase A2α inhibition (cPLA2α), which regulates arachidonic acid (AA) release and its subsequent synthesis (Payne et al., 2007; Ntranos et al., 2014). In 2007, Payne et al. found that fingolimod, but not fingolimod-P inhibits antigen-induced AA release and subsequently thromboxane and prostaglandin secretion in an independent way from S1P receptors (Figure 2). This inhibition of cPLA2α occurs at picomolar concentrations that stay within the range of FTY720 plasma concentrations, which is 30 nM at the steady-state level (Zemann et al., 2006). They concluded that fingolimod directly and specifically inhibits cPLA2α under *in vivo* and *in vitro* conditions (Payne et al., 2007). This direct anti-inflammatory action of fingolimod may expand its therapeutics uses, as it has been shown that cPLA2α inhibitors can alleviate collagen-induced arthritis (Tai et al., 2010; Feuerherm et al., 2019). cPLA2α is involved in cancer angiogenesis and tumorigenesis (Wen et al., 2013). As a result, cPLA2α inhibitor AVX235 has been shown to reduce vascularization and growth in breast cancer tumors (Kim et al., 2016), adding weight to the studies suggesting the feasibility of fingolimod use for cancer therapy. The cPLA2α inhibition can have several potential therapeutic benefits in inflammatory diseases, and we thus anticipate profound future interest in this area.

### Lysophosphatidic Acid Synthesis Reduction

Lysophosphatidic acid **(**LPA), produced by autotaxin, plays roles in different physiological and cellular processes like differentiation, proliferation, migration, survival, and pathological processes like inflammation and invasion of cancer cells (Valdés-Rives and González-Arenas, 2017). *In vitro*, fingolimod-P competitively inhibits autotaxin (IC_50_ = 0.3–0.4 µM) and orally administered fingolimod (3 mg/kg) reduces plasma levels of LPA in mice (van Meeteren et al., 2008). Through modulating LPA, fingolimod can promote peripheral nerve myelination (Szepanowski et al., 2016). Exploring the autotaxin/LPA axis may lead to the discovery of further fingolimod anticancer properties (van Meeteren et al., 2008).

### Protein Phosphatase 2A Activation

Fingolimod is also a well-known activator of Protein phosphatase 2 (PP2A) (Nagahara et al., 2001; Matsuoka et al., 2003), that plays a principal role as a regulator of cell cycle/division and growth, apoptosis, and regulation of various signal transduction pathways relevant to inflammation (Oaks and Ogretmen, 2015). The mechanisms of how fingolimod regulates PP2A are not fully known. However, disruption of interaction of PP2A to SET (endogenous inhibitor of PP2A) seems the most likely event (De Palma et al., 2019). Fingolimod binding induces chemical changes at the N-terminal residues of SET, making SET unavailable for dimerization or oligomerization. Fingolimod binding eventually separates SET from PP2Acα, thus leading to PP2A activation (De Palma et al., 2019). As depicted in Figure 2, fingolimod also reduces the inhibition of PP2A by the accumulation of SET into the nucleus, allowing PP2A to recover its activity in the cytoplasmic compartment (Pippa et al., 2014). Phosphorylation of the tyrosine residue Tyr307 causes Leu 309 methylation, resulting in PP2A inactivation (Clark and Ohlmeyer, 2019). Dephosphorylation of PP2A subunit C at Tyr307 site by fingolimod activates PP2A (Zhong et al., 2020). Additionally, fingolimod further dephosphorylates AMPKɑ at the Thr172 site, then decreases eEF2 and induces cell death in multiple myeloma cells (Zhong et al., 2020). This pharmacological event is believed to contribute to fingolimod’s general antitumor and anti-inflammatory. As noted before, PP2A activation by fingolimod causes activation of tristetraprolin (TTP), thus reducing inflammatory cytokines and improving neurological function, suppression of brain edema, and inhibiting apoptosis (Yin et al., 2018). Decreased phosphorylation of PP2A targets, Akt, and ERK 1/2 is also a consequence of PP2A activation by fingolimod attributed to anticancer manifestations in colorectal cancer (Cristóbal et al., 2014).

### Autophagy Modulation

Another fingolimod action is the induction of autophagy. In many cells, the autophagy markers such as microtubule-associated protein-1 light chain 3 (LC3) and Beclin1 are enhanced by fingolimod. Fingolimod-induced death of ovarian cancer cells is caspase-3 independent and includes cellular swelling and cytoplasmic vacuolization with apparent features of necrotic cell death and enhanced number of autophagosomes and LC3-II, and p62 degradation (Zhang et al., 2010). The caspase-independent cell death by fingolimod was also observed in lymphoblastic leukemia cells, accompanied by an increase in autophagosomes and LC3-II. However, since fingolimod-P also induced autophagy but not cell death, it was concluded that autophagy was pro-survival in that setting, and cytotoxic effects of the fingolimod were attributed to ROS induction (Wallington-Beddoe et al., 2011). Fingolimod also killed melanoma and myeloma cells by ROS enhancement. So, it was concluded that ROS acts as a regulator of fingolimod-induced apoptosis and autophagy (Liao et al., 2012; Li et al., 2013). In mantle cell lymphoma, fingolimod treatment affected autophagic flux in two ways: first, activation of upstream steps shown by the accumulation of autolysosomes and increased LC3-II, followed by disruption of autophagy at late stages (Alinari et al., 2011). In TBI, the acquired neuroprotection by fingolimod has been attributed to the enhancement in the expression of LC3 and Beclin 1, known as autophagy markers, and activation of the PI3/Akt pathway (Zhang et al., 2016). ROS-induced autophagy has been proposed as an antitumor effect of fingolimod in glioblastoma, oral squamous cancer cells, and ovarian cancer cells (Zhang et al., 2015; Bai et al., 2017). Fingolimod induces ROS-c-Jun N-terminal kinase-protein 53 (ROS-JNK-p53) loop-dependent autophagy, alongside apoptosis and necroptosis, in human glioblastoma cells. This autophagy is mediated by the PI3K/Akt/mTOR/p70S6K pathway (Zhang et al., 2015). However, in pancreatic stellate cells, fingolimod inhibited autophagy via suppressing AMPK and activating the mTOR pathway (Cui et al., 2019). It seems that the pharmacological effects produced by fingolimod under different pathological conditions do vary depending on the best-required outcome for the tissue. In an ischemic stroke model in mice, fingolimod dose-dependently decreased the induction of autophagosome proteins, LC3-II and, Beclin-1 leading to infarct volume reduction. Attenuated neuronal autophagy was shown to be mediated through the mTOR/p70S6K pathway and modulation of S1P signaling (Li et al., 2017). Accordingly, fingolimod modulation of autophagy is cell-type and context-dependent; so, depending on the context, autophagy functions are either pro-death or pro-survival (140). Presented in Table 1 are studies reporting both pro or anti survival effects of fingolimod under different pathological conditions.

**TABLE 1:** Fingolimod as an autophagy inducer/blocker and its role in cell death/survivals.

Author/Year	Model	Findings	Evidence
Zhang 2010	Ovarian cancer cells	↑ Autophagosomes and formation and accumulation of LC3-II	Pro-survival
Zhang et al. (2010)		↑Autophagic flux	
		↑LC3 turnover and p62 degradation	
Wallington-Beddoe 2011	Acute lymphoblastic leukemia Cell lines	↑Autophagosomes, LC3II expression ↑Autophagic flux	Pro-survival
Wallington-Beddoe et al. (2011)			
Liao 2011	Multiple myeloma (MM) cell line U266	↑LC3B-II	Pro-death
Liao et al. (2011)			
Romero Rosales 2011	Murine hematopoietic cell line FL5.12 and *In vivo*	↑LC3-II	Pro-survival
Romero Rosales et al. (2011)		↑Autophagosomes	
		↑Autophagic flux	
Alinari 2011	Mantle cell lymphoma (MCL) cell lines	Accumulation of autolysosomes and increased LC3-II and p62 levels	Pro-death
Alinari et al. (2011)		↑CD74	
Liao 2012	Multiple myeloma (MM) cell line U266	↑Conversion of LC3-I to LC3-II	Pro-death
Liao et al. (2012)		↑Autophagic flux	
Li 2013	Multiple myeloma cell line U266	↑LC3B-II	Pro-death
Li et al. (2013)			
Tay 2014	Human melanoma cells Mel-RM and MM200 cells	↑Conversion of LC3-I to LC3-II	Pro-death
Tay et al. (2015)		↑LC3-II	
		Degradation of sequestosome 1 (SQSTM1/p62)	
Zhang 2015	U251MG, U87MG, SHG44 and A172 glioblastoma cell lines and *In vivo*	↑Conversion of LC3-I to LC3-II	Pro-death
Zhang et al. (2015)		↑LC3-II	
		↑Beclin 1	
		↑Autophagosomes	
		↓p62/SQSTM1	
		↑Autophagic flux	
Ahmed 2015	Hepatocellular carcinoma cell lines Huh7 and HepG2	↑LC3-II	Pro-death
Ahmed et al. (2015)		↑p62	
Zhang 2016	Mouse model of TBI	↑LC3-II	Pro-survival
Zhang et al. (2016)		↑Beclin 1	
		↓p62	
Li 2016	Colorectal cancer cells	↑LC3B-II accumulation	Pro-death
Li et al. (2016)		↑Autophagosomes	
Bai 2017	Oral squamous cell carcinoma cellsSCC4, SCC25, and SCC2095	↑LC3B-II conversion	Pro-death
Bai et al. (2017)		↓p62	
		Accumulation of autophagosomes	
Li 2017	Ischemic brain stroke in mice	↓Induction of autophagosome proteins	Pro-survival
Li et al. (2017)		↓ LC3-II	
		↓Beclin 1	
Sun 2018	Foam cells	↑LC3II	Pro-survival
Sun et al. (2018)			
Cui 2019	Pancreatic stellate cells	↓ LC3B-II	Pro-death
Cui et al. (2019)		↓Atg5	
		↑p62	
Ota 2019	Non-small cell lung cancer cell line A549	↑ MAP1 LC3B-II	Pro-death
Ota et al. (2019)		Accumulation of SQSTM1	
		↓Autophagic flux	
Hu 2021	Primary microglia cells	↓LC3-II/LC3-I	Pro-survival
Hu et al. (2021)		↓Beclin 1	
		↑p62	
		↓Autophagic flux	

### Cell Cycle Arrest and Apoptosis Induction

Another significant pharmacological effect of fingolimod is the drug’s ability and the capacity to induce apoptosis in pathological conditions. The revelation began in 1996 when Suzuki et al. reported that fingolimod promptly induced lymphocyte apoptosis (Suzuki et al., 1996). Interestingly, it was later shown that fingolimod acts by homing T cells without affecting the total number of T cells (Chiba et al., 1998). However, its apoptotic inducing effect occurs in various cancer cells under *in vitro* and *in vivo* conditions. The reports include but are not limited to gastric cancer, breast cancer, renal cancer, pancreatic cancer, prostate cancer, multiple myeloma, different forms of leukemia, hepatocellular carcinoma, and glioma (White et al., 2016). The ability of fingolimod to induce apoptosis is mediated through multiple cell death signaling pathways. Apart from regulating S1P receptors, fingolimod induces G0/G1 cell cycle arrest and apoptosis in the human lymphoma cell lines HL-60 and Jurkat via affecting mitochondrial permeability transition and cytochrome c release (Nagahara et al., 2000). Modulation of mitogenic signaling, cell-cycle regulators, induction of G1 arrest, and apoptotic death are also reported in DU145 cells as androgen-independent prostate cancer cell lines (Permpongkosol et al., 2002). In T98G human glioma cells, fingolimod induced apoptosis through the focal adhesion kinase (FAK) dephosphorylation and cutting off the FAK-PI3-kinase pathway. Caspase-6 activation was responsible for apoptosis induction by fingolimod in these cells (Sonoda et al., 2001). Both intrinsic (caspase and mitochondrial-dependent) and extrinsic apoptotic pathways are involved in the apoptotic death triggered by fingolimod (Fujino et al., 2002; Zhang and Wang, 2020). Apoptotic activation of caspase cascade in fingolimod-treated Jurkat cells may be initiated by activation of mitochondria (Fujino et al., 2001). Other most fingolimod noted apoptotic pathways are inactivation of ERK/MAP kinase (Estrada-Bernal et al., 2012), inhibition of PI3K/Akt/mTOR signaling pathway and subsequent reduction in phosphorylated p70S6k levels, caspases-3, and 9 activations (Estrada-Bernal et al., 2012), cleavage of Poly (ADP-ribose) polymerase (PARP), MMP loss (Zhao et al., 2018), down-regulating anti-apoptotic proteins Mcl-1, BCl-2, and cleavage of Bid and Bim (Kiyota et al., 2013). An increase in phosphatase and tensin homolog (PTEN), which inhibits pAkt and murine double minute 2 (MDM2), followed by increased p53 (Zheng et al., 2010), and ceramide levels and activation of PP2A (Chen et al., 2014) are also included. However, the complete and exact mechanisms behind the apoptotic properties of fingolimod remain to be determined through future studies. Interestingly, an anti-apoptotic role of fingolimod has also been reported in other pathological conditions such as brain injury, where the inhibition is highly beneficial in the process of treatment. Here, fingolimod reduced apoptosis following brain insults shown by the increase in Bcl-2, Bcl-xL, and decrease in the cleaved caspase-3 and cytoplasmic cytochrome c expression (Zhang et al., 2016; Yin et al., 2018). A summary of the main molecular targets of fingolimod is presented in Table 2.

**TABLE 2:** Molecular targets of fingolimod and its relevant effective concentrations/doses.

Author/Year	Model/Therapeutic dose	Molecular target/Form	Effect	Mechanisms of action
Chiba 1998	Rats	-	Decreases Circulating Lymphocytes	Lymphocyte homing acceleration
Chiba et al. (1998)	0.1–1 mg/kg oral			
Mandala 2002	Mice and Rats	S1P receptors/Phosphorylated	Rapid peripheral lymphopenia	Lymphocytes sequestration
Mandala et al. (2002)	2.5 mg/kg IV			
Brinkmann 2002	Rats	S1P receptors	Decreases Circulating Lymphocytes	Lymphocytes sequestration in secondary lymphatic tissues and away from inflammatory lesions and graft sites
Brinkmann et al. (2002)	0.1–1 mg/kg oral	(1,3–5)/Phosphorylated		
Sanchez 2003	Mice	S1P receptors/Phosphorylated	Decrease in VEGF-induced vascular permeability, maintains the integrity and functionality of endothelial cells	stimulates VE-cadherin and *ß*-catenin translocation and assembly into cell-cell junctions
Sanchez et al. (2003)	50 µg by gavage			
Matloubian 2004	Mice	S1P1/Phosphorylated	Lymphopenia	S1P1 downregulation
Matloubian et al. (2004)	1.1 or 1 mg/kg IP			
Bandhuvula 2005	Mice	S1P lyase/Non- Phosphorylated	Lymphopenia	S1P lyase inhibition
Bandhuvula et al. (2005)	1 mg IP			
Lamontagne 2006	Mice	S1P1/Phosphorylated	Inhibition of tumor-associated angiogenesis	S1P1 internalization
LaMontagne et al. (2006)	0.3 or 3 mg/kg oral			
Payne 2007	*In vitro*	cPLA2α/Non- Phosphorylated	Inflammation inhibition	cPLA2α inhibition
Payne et al. (2007)	200–800 p.m.			
Schmid 2007	Mice	S1P1/Phosphorylated	Inhibition of tumor-associated angiogenesis	
Schmid et al. (2007)	10 mg/kg IP			
Toneli2010	*In vitro*	SK1/Non-Phosphorylated	Induces apoptosis in cancer cells	ubiquitin-proteasomal degradation
Tonelli et al. (2010)	50 µM			
Lahiri 2009	*In vitro*	Ceramide synthase/Non-Phosphorylated	-	noncompetitive inhibition toward acyl-CoA and sphinganine
Lahiri et al. (2009)	25–100 µM			
Chen 2013	Rats	Ceramide synthase/Non-Phosphorylated	Protects retina from light-induce degeneration	De novo Ceramide synthase inhibition
Chen et al. (2013)	10 mg/kg IP			
Dawson 2011	*In vitro*	ASMase/Non-Phosphorylated	-	proteolytic degradation of the enzyme complex
Dawson and Qin, (2011)	10 µM			
Hait 2014	*In vitro*	class I HDACs/Phosphorylated	facilitates fear extinction memory reactivates ERα expression	Binding to active site of class I HDACs leading to enzymatic activity inhibition
Hait et al. (2014)	5 µM			
Hait 2015	Mice			
Hait et al. (2015)	1 mg/kg oral			
Segura-Ulate 2017	*In vitro*	HDAC/-	reverses *a*-synuclein-induced downregulation of BDNF	increased histone 3 acetylation
Segura-Ulate et al. (2017)	150 nM			
Perla 2020	*In vitro*	HDAC/-	induces antitumor activities in medulloblastoma cells	increased histone 3 acetylation
Perla et al. (2020)	7.5 or 10 µM			
Ji 2019	Rat	HDAC/Phosphorylated	M1 to M2 shift decrease pro-inflammatory factors prevent ischemia-induced brain injury	prevents KLF4 to interact with HDAC1
Ji et al. (2019)	2 mg/kg IP			
Qin 2013	*In vitro*	TRPM7/Non-Phosphorylated	inhibits cell proliferation and migration	TRPM7 inhibition
Qin et al. (2013)	1 µM			
Schilling 2014	*In vitro*	TRPM7/-	inhibits cell proliferation and polarization of macrophages	TRPM7 inhibition
Schilling et al. (2014)	3 µM			
Van meeteren 2008	*In vitro*	Autotaxin/LPA axis/Phosphorylated	reduces plasma levels of LPA	Autotaxin inhibition
van Meeteren et al. (2008)	100–250 nM			
	Mice			
Szepanowski 2016	1 mg/kg oral			
	Mice			
Szepanowski et al. (2016)	1 mg/kg IP	Phosphorylated	LPA reduction	LPA synthesis inhibition
Matouska 2003	*In vitro*	PP2A/Non-Phosphorylated	Akt and p70S6k/p85S6k dephosphorylation leading to cell apoptosis	disruption of interaction of PP2A to SET, leading to PP2A activation
Matsuoka et al. (2003)	2.5–10 µM			

## Therapeutic Applications

As discussed above, fingolimod affects several processes and cellular signaling pathways, making it suitable for use in diverse pathological conditions. Besides its clinical efficacy in MS, under its immunomodulatory effect and depletion of peripheral lymphocytes, the drug has been nominated as a potential therapy for other immune-related diseases. However, therapeutic indications of fingolimod are not just confined to immune-related diseases. The following sections summarize fingolimod’s most studied potential therapeutic uses, ranging from immune-related diseases to CNS injuries and cancer.

### Immune-Mediated Diseases

#### Multiple Sclerosis

The only FDA-approved therapeutic use of fingolimod is for treating MS. Generally considered an autoimmune disease; it is long believed that the entering of autoreactive T cells into the CNS ignites inflammatory responses resulting in demyelination and axon loss (Lubetzki and Stankoff, 2014). In 1998, Chiba et al. reported that fingolimod acts through lymphocyte (mainly T cells) sequestration into main lymphoid organs by the acceleration of lymphocytes homing (Chiba et al., 1998) inducing systemic lymphopenia, and also by inhibiting T cell functions (Luo et al., 1999). Further investigations suggested that the drug acts differently from classical immunosuppressants (mainly calcineurin inhibitors) and suppresses *in vivo* immune functions mainly by acting on GPCRs (Brinkmann and Lynch, 2002; Mandala et al., 2002). With more knowledge about fingolimod mechanism of action, several *in vivo* preclinical experiments evaluating fingolimod efficacy in animal models of MS were conducted between 2002 and 2006 (Brinkmann and Lynch, 2002; Rausch et al., 2004; Kataoka et al., 2005; Kappos et al., 2006), confirming that inhibition of T-cell responses or their migration into the CNS plays a significant role in the anti-inflammatory effect of fingolimod (Volpi et al., 2019). The studies have been further expanded, thus revealing more information on the activity of the drug. In an attempt to reproduce the inflammatory pathology of MS, scientists widely use EAE. Magnetic resonance imaging (MRI) and histological assessments showed that in the EAE model, oral fingolimod preserved inflammatory lesions and improved neurologic function in the rat’s central nervous system (Fujino et al., 2003; Rausch et al., 2004). In 2007, Balatoni et al. reported that pretreatment with fingolimod (0.4 mg/kg) prevented distractions to the visual and somatosensory evoked potentials as symptoms of the EAE. Treatment from day 25–45 inhibited EAE induced paralysis development and normalized the electrophysiological responses, alongside brain and spinal cord demyelination decrease (Balatoni et al., 2007). These encouraging results suggested fingolimod as a promising candidate for clinical studies in the treatment of MS. Subsequent successful trials proved the efficacy of fingolimod in the management of RRMS (Kappos et al., 2006; Cohen et al., 2010; Kappos et al., 2010). Eventually, in September 2010, fingolimod was approved by the FDA for use to treat relapsing-remitting MS. In May 2018, FDA extended the fingolimod approval to include the treatment of pediatric RRMS. Since FDA approval, noticeable information regarding the various effects of the drug in the management of MS has been generated. In a model of EAE, it was found that fingolimod efficacy is way more than immunological effects on lymphocytes and requires astrocytes S1P1 modulation (Choi et al., 2011). What is now certain is that fingolimod possesses more than just anti-inflammatory effects in MS (Yazdi et al., 2020). There are some conflicting data about fingolimod’s ability to reduce anxiety-like behaviors in EAE models of MS. While It was previously shown that fingolimod reduces anxiety-like behavior (Bonfiglio et al., 2017), a new study fails to show anxiety-like symptoms in EAE mice models of MS (Kocovski et al., 2021). Mounting evidence suggests that fingolimod also has neuroprotective activity (Slowik et al., 2015; Sternberg et al., 2018; Yang et al., 2021) and acts as a myelin regeneration booster by affecting neural precursor cells and oligodendrocytes lineage (Coelho et al., 2007; Miron et al., 2010; Heinen et al., 2015; Qin et al., 2017). Our published studies show that fingolimod enhances oligodendrocytes lineage markers in EPI-NCSCs *in vitro* (Pournajaf et al., 2020) and *in vivo* in neural precursor cells to participate in myelin repair (Yazdi et al., 2015). We also found that following fingolimod administration in an animal model of demyelination induced by local injection of lysolecithin, inflammatory indices are reduced, and remyelination is enhanced (Yazdi et al., 2015).

#### Other Immunomodulatory Diseases

T-cells are considered the culprits of many other autoimmune diseases, including diabetes mellitus, psoriasis, systemic lupus erythematosus (SLE), and rheumatoid arthritis (RA). Consequently, S1P receptor modulators may have broader beneficial therapeutic effects worth considering. Indeed, preclinical surveys have found that fingolimod can halt the development of different models of RA by acting on the S1P signaling pathway (Yoshida et al., 2016; Zhu et al., 2021), neuropsychiatric SLE (Mike et al., 2018), and also ameliorate clinical and histological signs of psoriasiform dermatitis (Okura et al., 2021). Fingolimod is considered a treatment to prevent diabetes development by preserving *ß*-cell mass (Maki et al., 2005; Moon et al., 2013). However, there are no published reports on the clinical efficacy of fingolimod in diabetes yet. Furthermore, it has been recently revealed that fingolimod may act as a prophylactic therapy through humoral immune response regulation and alleviate experimental autoimmune myasthenia gravis (EAMG). On this basis, it has been nominated as an adjunct pharmacological therapy in managing myasthenia gravis (Liu et al., 2021). In addition, other immune-related diseases like inflammatory bowel disease have shown promising results following the use of more selective S1P receptor modulators rather than fingolimod (Pérez-Jeldres et al., 2021).

### CNS Injuries

#### Brain Injury

Fingolimod has protective effects in various kinds of brain injuries, including ischemic stroke, intracerebral hemorrhage, and TBI. Additionally, through suppressing both neuronal apoptosis and autophagy, the drug exerts beneficial therapeutic effects after stroke. This phenomenon is thought to be aided by the anti-inflammatory mechanisms rather than direct effects on neurons (Wei et al., 2011). In a study performed by Kraft et al., in 2013, they compared the effect of 1 mg/kg fingolimod on the ischemic stroke in wild type and Rag1^−/−^ mice (that lack T cells and B cells and are profoundly protected from ischemic neurodegeneration in the transient middle cerebral artery occlusion (tMCAO) model). They found that fingolimod caused more minor strokes and improved functional outcomes in wild-type mice. However, it failed to reduce infarct volume or improve function in Rag1^−/−^ mice (Kraft et al., 2013). They also showed that fingolimod could diminish thrombosis formation and microvascular dysfunction. They concluded that lymphopenia induction and consequent microvascular thrombosis reduction are the principal fingolimod effects in stroke (Kraft et al., 2013). Since fingolimod reduces platelet aggregation and some coagulation parameters, it has been proposed as an adjunct treatment in ischemic conditions (Zhao Z. et al., 2017). In different models of stroke and TBI, alongside a decrease in circulating lymphocytes, fingolimod reduces inflammatory cytokines like TNFα, IL-1β and enhances anti-inflammatory cytokines including IL-6 and TGFβ (Liesz et al., 2011; Dong et al., 2018; Ji et al., 2019). Fingolimod attenuated early accumulation of endothelial-monocyte activating polypeptide II (EMAP-II (+) and major histocompatibility complex class II (MHC-II (+) reactive monocytes following TBI, that candidate the drug to inhibit brain inflammatory response after TBI (Zhang et al., 2007). Activating PP2A by fingolimod leads to dephosphorylation and activation of mRNA-destabilizing protein tristetraprolin and reduces the production of TNF-α, IL-6, and IL-8 in early brain injury (Yin et al., 2018). Anti-inflammatory effects of fingolimod (0.5 mg/kg) after TBI or ischemic stroke also occurs via restoration of the neurovascular unit by decreasing endothelial cell apoptosis and attenuating the activation of astrocytes (Cheng et al., 2021) or preventing the tight junction protein redistribution (Wang Z. et al., 2020). Attenuation of iron deposition is also an outcome of fingolimod use in intracerebral hemorrhage (Yang et al., 2019). Inactivation of microglia/macrophage or modulating microglia toward M2 polarization via STAT3 pathway also has been taken into account for fingolimod beneficial effects in models of stroke or TBI (Czech et al., 2009; Moon et al., 2015; Gao et al., 2017; Qin et al., 2017). S1PR1 activation is another proposed mechanism for reducing neuronal injury after ischemic stroke in rats (Hasegawa et al., 2013). However, although experimental stroke prognosis improves by fingolimod activation of S1P receptors, this is not the only protective effect of fingolimod (Hasegawa et al., 2017). However, there are also doubts about the effectiveness of fingolimod treatment in the brain injury context. Herz et al., in 2018 reported that a single injection of fingolimod (1 mg/kg) exacerbates hypoxic-ischemic brain injury in neonatal rats. Concomitant with the increase in the infiltration of innate immune cells, fingolimod significantly reduced cerebral infiltration of CD4 T cells, leading to provoked brain injury (Herz et al., 2018). Parts of neuroprotective effects of fingolimod after TBI are through the activation of the PI3K/Akt pathway and autophagy (Zhang et al., 2016). How fingolimod regulates PI3K/Akt pathway is not clearly known. It has been reported that phosphorylated fingolimod acts through S1PR3 to inhibit the TLR2/4-PI3K-NFκB signaling pathway (Dong et al., 2018). Affecting PTEN as PI3K/Akt regulator is another proposed mechanism (Zhang and Wang, 2020), yet further investigations have to be performed to unravel the exact mechanism. The promising results of fingolimod in different models of brain injury led to its use in clinical trials. A clinical trial in acute ischemic stroke (NCT04675762) showed that 3 days combination of 0.5 mg fingolimod with alteplase at the very first hours of ischemic stroke onset diminished reperfusion injury. The decrease in reperfusion injury was parallel with improving patients’ clinical outcomes (Zhu et al., 2015). Currently, two other clinical trials evaluate the effects of fingolimod in endovascular treatment for acute ischemic stroke (NCT04629872) and as a treatment for cerebral edema after intracerebral hemorrhage (NCT04088630). Presented in Table 3 is a summary of fingolimod mechanisms of action in various experimental models of brain injury.

**TABLE 3:** Summary of studies evaluating the effect of fingolimod in brain injuries.

Author/Year	Model	Molecular findings	Histologic and clinical findings	Proposed mechanisms of action
Zhang 2007	Traumatic brain injury (TBI) (weight drop)	↓EMAP-II+ and MHC-II + monocytes	-	-
Zhang et al. (2007)				
Zhang 2008	TBI (Weight drop)	↓IL16(+) cells	-	-
Zhang et al. (2008)				
Shichita 2009	Cerebral ischemia-reperfusion	↓ Infiltrating T lymphocytes	↓Infarct volume	-
Shichita et al. (2009)		No change in macrophage infiltration		
Czech 2009	Focal cerebral ischemia	↓Neutrophils	↓lesion size	-
Czech et al. (2009)		↓Activated macrophage/microglia	↑Neurologic function	
		↓Circulating blood leukocytes	↓apoptotic cell death	
Hashegawa 2010	Ischemic stroke	↑Akt and ERK-1 phosphorylation	↓Infarct volume	Activation of Akt and ERK via S1PR1, which prevented apoptosis
Hasegawa et al. (2010)		↑Bcl2	↑Neurologic function	
		↓Cleaved caspase 3		
Wei 2011	Focal cerebral ischemia	↓Activated macrophage/microglia	↓ Edema	Fingolimod might decrease tissue damage by limiting the levels of cytotoxic agents, rather than by a direct neuroprotective effect
Wei et al. (2011)		↓Inflammation	↓Infarct size	
		↓Neutrophil infiltration	↓Neurological deficit	
		↓ICAM-1-positive blood vessels	↓Brain water content	
			↓Apoptotic cell death	
Leisz 2011	Permanent and transient cortical ischemia	↓Lymphocyte brain invasion	No change in infarct volume and behavioral dysfunction	-
Liesz et al. (2011)		↓IL-1β and IFN-γ		
		↑IL-6 and TNF-α		
Rolland 2011	Intracerebral hemorrhage (collagenase)	-	↓Brian edema	-
Rolland et al. (2011)			↑Neurological function	
Pfeilschifter 2011	Ischemic stroke (tMCAO)	-	↓Lesion size	Fingolimod does not aggravate immune depression after stroke despite reducing number of circulating leukocytes
Pfeilschifter et al. (2011)			↓pulmonary infections	
Rolland 2013	Intracerebral hemorrhage (collagenase)	↓Lymphocytes	↑Neurological function	Fingolimod reduced cerebral inflammation by reducing brain infiltration of T-lymphocytes
Rolland et al. (2013)		↓ (ICAM-1), (INF-γ), and(IL-17)	↓Brain edema	
			↓Brain atrophy and neuronal cell death	
Brunkhorst 2013	Photothrombotic stroke	↓Reactive astrogliosis	↑Functional outcomes	-
Brunkhorst et al. (2013)		↑Postsynaptic densities		
		↑ VEGFα		
Campos 2013	Thromboembolic stroke (MCAO)	↓Hemorrhagic transformation (in combination with tissue Plasminogen Activator)	↓Infarct volume	-
Campos et al. (2013)			↓Neurological deficits	
Kraft 2013	Ischemic stroke	↓Lymphocyte circulation	↓Stroke size	Lymphocytopenia induction
Kraft et al. (2013)		↓Microvascular thrombosis	↑Functional outcome	
			↑Cerebral perfusion	
Hashegawa 2013	MCAO	↓S1PR1 expression on neurons	↑Neurological function	Fingolimod reduced neuronal injury possibly via S1PR1 activation
Hasegawa et al. (2013)			↓Infarct volume	
Mencl 2014	TBI (Focal cortical cryolesion)	↓Circulating lymphocytes	No change in lesion size, functional outcomes, and BBB disruption	-
Mencl et al. (2014)				
Lu 2014	Intracerebral hemorrhage (collagenase)	No change in CD68 (a marker for macrophage and microglia)	↓Edema, apoptosis and brain atrophy	Protective effects of fingolimod may involve mechanisms other than inflammation
Lu et al. (2014)			↑Neurologic function	
Moon 2015	MCAO	↓Microglial activation and astrogliosis	-	-
Moon et al. (2015)		↓ TNF-α		
Schuhmann 2016	tMCAO	No change in astrogliosis, BDNF expression, and synaptogenesis	↓Infarct volume	Key mode of fingolimod action in stroke is the reduction of microvascular thrombosis
Schuhmann et al. (2016)			↓Motor deficits	
Schlunk 2016	Intracerebral hemorrhage	No change in MMP-9	No change in mortality,neurological outcomes, and edema	Fingolimod has no beneficial effects in the acute phase of experimental ICH
Schlunk et al. (2016)				
Nazari 2016	MCAO	↑ LTP magnitude without any effects on presynaptic plasticity and neurotransmitter release probability	↓ Lesion volume	Fingolimod improved the memory performance after MCAO by LTP induction via post-synaptic mechanism
Nazari et al. (2016)			↑Memory	
Zhang 2016	TBI (weight drop)	↓Cleaved caspase 3, PARP, Bax and cytochrome C	↑Neurobehavioral function	Fingolimod reduced TBI neuronal apoptosis via Activating modulation of PI3K/Akt and autophagy
Zhang et al. (2016)		↑Bcl-2 and Bcl-xL and mitochondrial cytochrome C	↓Brain edema	
		↑Phospho-Akt	↓Apoptotic cell death	
		↑LC3-II and Beclin 1		
		↓p62		
Gao 2017	TBI (controlled cortical impact injury	↓Infiltrated T lymphocytes and NK	↑Neurological functions	Fingolimod administration extensively modulates multiple immuno-inflammatory responses
Gao et al. (2017)		↑percentage of regulatory T (Treg) cells and IL-10	↓Brain edema	
		↑M2/M1 microglia	↓BBB damage	
		↓Inflammatory cytokines		
Liu 2017	TBI (Weight drop)	↓Micro vesicle	↓Apoptotic neuron death	-
Liu et al. (2017)		↓ amoeboid-like cells with P2X7R-ir	↑ Neurobehavioral outcomes	
		↓ IL-1β		
		↓Phosphorylated p38		
		↓GFAP-ir cells		
Rolland 2017	Neonatal germinal matrix hemorrhage	↑ ZO1, Occludin, and Claudin-3 Expression	↑long-term neurocognitive performance and ↓brain tissue loss	Fingolimod treatment tempered acute post-hemorrhagic BBB disruption via the activation of the S1PR1/Akt/Rac1 pathway
Rolland et al. (2017)		↑Akt phosphorylation	↓Brain water content	
		↑Rac activation		
Hashegawa 2017	Subarachnoid hemorrhage	-	↓Neurological deficits	Fingolimod reduction of injury was associated with pleiotropic actions of the drug
Hasegawa et al. (2017)			↓Brain edema	
Qin 2017	White matter (WM) ischemic injury (bilateral carotid artery stenosis)	↓Microglial activation	↓Cognitive decline ameliorate the disruption of Ranvier’s nodes	Fingolimod modulated microglia toward M2 polarization via STAT3 pathway
Qin et al. (2017)		↑ Oligogenesis and OPCs maturation		
		↓IL-1β and TNF-α	↓OPC apoptosis	
		↑IL-13 and TGF-β	↑Oligodendrocytes survival and differentiation	
Li 2017	Ischemic stroke	↓LC3-II and Beclin1	↓infarct volumes ↓neuronal apoptosis	Fingolimod suppresses neuronal autophagy through the mTOR/p70S6K pathway
Li et al. (2017)		↑mTOR and p70S6K	↓Functional deficits	
Herz 2018	Hypoxic-ischemic (HI) brain injury	↓ CD4 &CD8 Tcells	↑Brain tissue injury	Peripheral T Cell depletion by fingolimod Exacerbates hypoxic-ischemic brain injury in neonatal mice
Herz et al. (2018)		↓MAP2 and MBP		
Dong 2018	*In vitro* model of cerebral ischemia and reperfusion injury, oxygen-glucose deprivation (OGD)	↓HMGB1 &TNF-α	-	Fingolimod acts on S1PR3 to regulate the inflammatory cascades via inhibiting PI3K/NFκB signaling pathway
Dong et al. (2018)		↓TLR2		
		↓PI3K phosphorylation		
		↓NF-κB activation		
Salas-Perdomo 2019	Ischemia/reperfusion	↓lymphocyte infiltration	-	Fingolimod attenuated HT after cerebral ischemia/reperfusion in a lymphocyte-independent fashion
Salas-Perdomo et al. (2019)		↓β-catenin degradation		
		No change in Evans blue extravasation		
Shang 2020	Photothrombotic (PT) Ischemic stroke	↓CD16 and iNOS	↓Neuronal loss	Fingolimod treatment could skew microglial polarization directly to the M2 phenotype
Shang et al. (2020)		↑ CD206 and Arg-1	↑Motor function	
Li 2020	TMCAO in diabetic mice	↓ZO-1	↓Mortality rate	Due to negative impact of fingolimod on BBB integrity, it should be used with caution for ischemic stroke with diabetic comorbidity
Li et al. (2020b)		↓Occludin	No change in neurological score and infarct volume	
		↓S1PR1 protein levels	↑Brain edema	
		↑ Bcl-2/Bax Ratio		
		↓TNFα		
Wang 2020	tMCAO	↓ Iba1	↓Mortality	Fingolimod protected BBB integrity by preventing the redistribution of lamellipodia-located tight and adherens junctions into the cytoplasm via S1PR1 receptor signaling
Wang et al. (2020b)		↓ CD68-positive macrophages	↓Infarct Size ↑Functional Recovery	
		↑ZO-1 and VE-cadherin proteins ate cells lamellipodia	↓Apoptotic cell death	
		↑ ERK1/2	↓Neuroinflammation	
Wang 2020	Subarachnoid Hemorrhage (SAH)	↓IL-6 and TNFα	↑Neurologic function	-
Wang et al. (2020a)		↑IL-10 &TGF-β1	↓Brain water content	
		↑Treg cell		
		↓NK cells		
Diaz Diaz 2021	Intracerebral hemorrhage (collagenase)	↓ Circulating lymphocytes (CD3^+^, CD4^+^, and CD8^+^)	↑Survival	-
Diaz Diaz et al. (2020)			No change in lesion size and functional outcomes	
Cheng 2021	TBI (Weight drop)	↑Occludin and claudin-5	↓Endothelial cell apoptosis	-
Cheng et al. (2021)		↓ERK1/2	↑Neurologic function	
		↓S1PR1	↑Survival rate	
		↓Activated microglia and astrocytes	↑Neurologic function	
			↓BBB breakdown	

#### Spinal Cord Injury

With the knowledge of the immunomodulatory actions of fingolimod, its effects in spinal cord injury (SCI) have also been explored. Inflammation is a pivotal component of secondary injury following SCI, with numerous cells involved as mediators, including astrocytes, resident microglia, infiltrating immune cells, and endothelial cells (Hausmann, 2003). The release of inflammatory cytokines in or near the SCI site activates the immune cells and triggers them to move toward the lesion area, ultimately inducing an inflammatory response (Jones and Ren, 2016). The use of fingolimod treatment led to a drastic reduction of T-cell infiltration into the SCI lesion site at four and 7 days post-injury while not influencing other inflammatory cell populations. In the treated animals, better functional performance was following higher white matter sparing at the lesion epicenter. Improved bladder function and lower incidence of hemorrhagic cystitis were also observed compared to the control group (Lee et al., 2009). In another study, after rat SCI, functional recovery improved after combining 0.5 mg/kg fingolimod and tacrolimus. The combination therapy could also enhance electrophysiological results evaluated via amplitude and latency of somatosensory evoked potentials (Zhang et al., 2009). In a study by Norimatsu et al., fingolimod improved motor function after SCI and induced lymphopenia, reduced vascular permeability, T-cell infiltration, and astrocyte accumulation into the injury site while not affecting inflammatory cytokines, highlighting the immune-independent actions of fingolimod (Norimatsu et al., 2012). In another study, it was shown that fingolimod reduces astrocyte migration and proliferation. After SCI, local delivery of fingolimod with polycaprolactone (PCL) membrane attenuated neuron loss, reduced astrocytes accumulation, and lymphocytes infiltration into the injury site leading to functional recovery (Wang et al., 2015). Neuropathic pain, systemic and local inflammation, size of the damaged area, and astrogliosis after SCI was significantly reduced by 1.5 mg/kg fingolimod, administered 24 h after injury, resulting in motor recovery. Induction of lymphopenia and preservation of BBB are also linked to fingolimod’s beneficial effects in enhancing survival and allodynia amelioration (Yamazaki et al., 2020). However, unlike brain injuries, the number of studies evaluating the effects of fingolimod in SCI are scarce, so the underlined mechanisms of fingolimod action are not yet fully understood.

### CNS Diseases

#### Alzheimer’s Disease

As the multimodal abilities of fingolimod and its efficacy and safety in MS are well established, its use in other neurodegenerative diseases such as Alzheimer’s disease **(**AD) has been considered. In AD, where insoluble fibrillar amyloid *ß* (Aβ) containing plaques are considered the main culprits, known as the most common cause of dementia pathogenesis, S1P metabolism and signaling imbalance are also involved (Angelopoulou and Piperi, 2019). S1P activation has been shown protective in different AD models by preserving neurons and counteracting AD’s induced memory loss (Asle-Rousta et al., 2013; Hemmati et al., 2013; Takasugi et al., 2013). We have previously shown that chronic administration of 1 mg/kg fingolimod for 14 days causes significant mitigation in learning and memory loss induced by Aβ (42) and preserved hippocampal neurons from damage (Asle-Rousta et al., 2013). The beneficial effects of fingolimod in the AD context were attributed to central S1P modulation and downregulation of ceramide metabolism genes (Asle-Rousta et al., 2014; Joshi et al., 2017; Jęśko et al., 2020). However, in the report by Takasaki et al., a decrease in Aβ production in cultured neurons was S1PR1 and Gi independent (Takasugi et al., 2013). Upregulation in neurotrophic factors is one of the mechanisms behind the neuroprotective effects of fingolimod in AD (Fukumoto et al., 2014). In 2013, Doi and others found that fingolimod-P can suppress Amyloid β–induced neurotoxicity in primary mouse cortical neurons by enhancing BDNF expression and acting on TrkB/ERK1/2 signaling pathway (Doi et al., 2013). Recently, it has been shown that with the aid of BDNF, 10 nM fingolimod can modulate dysregulated dendritic architecture in primary hippocampal neurons (Patnaik et al., 2020). Moreover, fingolimod can downregulate AD’s neuroinflammation by reducing activated microglia, astrocytosis, and plaque density (Aytan et al., 2016; Kartalou et al., 2020). Anti-neuroinflammatory effects of the fingolimod on microglia and astrocytes also can rescue synaptic pathology in AD (Kartalou et al., 2020). Another mechanism for fingolimod reduction in Aβ neurotoxicity is the relocation of neurotoxic NMDA receptors from the extra-synaptic area to synapses and altering their ratio. This ratio change leads to a decrease in neuronal calcium responsiveness to neurotoxic soluble Aβ 1–42, making neurons resistant to changes of calcium homeostasis (Joshi et al., 2017). Since the lower doses of fingolimod (0.03 mg/kg/day) in an AD model in mice could also rescue memory loss and reduce activated microglia/astrocytes alongside the restoration of hippocampal GABA levels without inducing lymphopenia, it was suggested that therapeutic benefits of fingolimod are not related to lymphopenia (Carreras et al., 2019). In CNS tissue affected by AD, PP2A activity is reduced to 50% (Clark and Ohlmeyer, 2019). So, the PP2A activation ability of fingolimod may also be considered a therapeutic clue in AD. The accumulating data suggest the potential beneficial effects of fingolimod in AD treatment.

#### Parkinson’s Disease

Fingolimod can also attenuate 6-hydroxydopamine or rotenone-induced neurotoxicity used as models to simulate Parkinson’s disease **(**PD) pathology. Fingolimod (0.5 mg/kg) reduced neuroinflammation and preserved motor function by reducing apoptosis, dampening astrogliosis, and enhancing ERK 1/2 signaling phosphorylation (Zhao P. et al., 2017; Ren et al., 2017). Enhanced BDNF and S1PR1 expression are also involved in the fingolimod neuroprotection (Vidal-Martinez et al., 2019; Pépin et al., 2020). In a 1-methyl-4-phenyl-1,2,3,6-tetrahydropyridine (MPTP) mouse model of the PD, 1 mg/kg fingolimod also exerts neuroprotection and enhanced motor function related to the S1PR/Akt kinase signaling pathways (Motyl et al., 2018). Investigations revealed that upregulation of protective micro RNAs (miRNAs) is another proposed mechanism of action for fingolimod. These miRNAs can downregulate alpha-synuclein, reduce apoptosis and thus be beneficial in alleviating PD complications (Vargas-Medrano et al., 2019). Fingolimod (2 mg/kg) has been reported to reduce microglial activation induced by MPTP *in vivo* and in 1-methyl-4-phenylpyridinium (MPP) treated BV-2 microglial cells. Moreover, in MPP + -treated BV-2 cells and primary microglia, fingolimod caused a significant reduction in phosphorylation of PI3/K/Akt/GSK3β signaling pathway, reduced ROS generation and p65 phosphorylation by inhibition of NLRP3 inflammasome indicating potential strategy against PD (Yao et al., 2019). However, there is no consensus about the neuroprotective functions of fingolimod, as Koming et al. reported there was no neuroprotection after fingolimod pretreatment in an MPTP model of Parkinson’s disease, nor was there any sustainable BDNF enhancement (Komnig et al., 2018).

#### Epilepsy

With proven benefits in preserving neurons and BBB integrity and the ability to reduce inflammation, fingolimod was investigated as a potential therapy for epilepsy (Bascuñana et al., 2020). Gao et al. were the first to report that 1 mg/kg fingolimod has anti-inflammatory and antiepileptogenic effects in a lithium-pilocarpine experimental model of epilepsy. In fingolimod-treated rats, reduced IL-1β and TNFα levels in the hippocampus were accompanied by decreased incidence, duration, frequency, and severity of spontaneous convulsions (Gao et al., 2012). They later showed that through S1PR1 inhibition, 1 mg/kg fingolimod reduces seizure-induced P-glycoprotein (P-gp) overexpression (Gao et al., 2018). We found that fingolimod enhances remyelination in the pentylenetetrazol-induced kindling model, along with reducing hippocampal neuronal death (Gol et al., 2017). In an animal model of genetic absence epilepsy, fingolimod (1 mg/kg) showed transient antiepileptic effects and longer-lasting anti-cognition decline (Leo et al., 2017). During the chronic epileptic phase of the mouse kainate model, 6 mg/kg of the drug also showed neuroprotective and anti-gliotic effects besides reducing seizure frequency (Pitsch et al., 2019). Fingolimod’s ability to reduce seizure severity, prevent anxiety-like behavior, and reverse cognition impairment has also been shown in a more recent study in a rat model of the hypoxia-induced seizure (Najafian et al., 2021). Based on the cumulated experimental evidence so far, it would certainly be worth investigating the potential benefits of fingolimod as adjuvant therapy in drug-resistant epilepsy.

#### Other CNS Diseases

As neuroprotective and anti-inflammatory effects of fingolimod emerge, the drug proposed as a potential treatment in diverse neurologic diseases, including Huntington’s (Di Pardo et al., 2014), Amyotrophic lateral sclerosis (ALS) (Potenza et al., 2016), Rett syndrome (Deogracias et al., 2012), and schizophrenia (Francis et al., 2020). The phase 2 clinical trial for the effects of fingolimod on schizophrenia (NCT01786174) has been completed. For ALS, The results of a phase IIa trial of fingolimod demonstrated short-term safety and tolerability of 0.5 mg/day (Berry et al., 2017). However, it remains to be seen whether further clinical trials will eventually lead to the drug’s approval in ALS.

### Cancer

**FIGURE 3:**
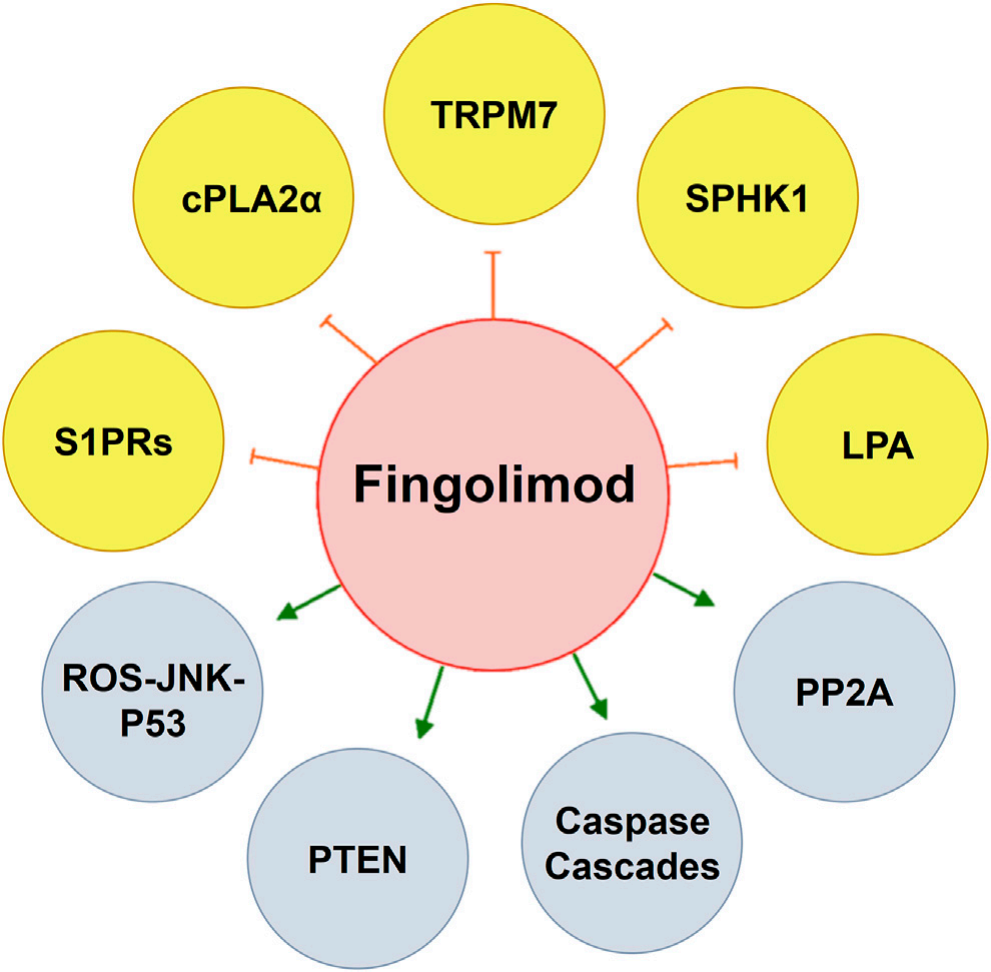
By modulating key pathways in oncogenesis, fingolimod has the potential use for cancer therapy. Fingolimod activates PP2A that plays a principal role as a regulator of cell cycle/division and growth. Fingolimod can induce apoptotic pathways by activation of caspase cascades, enhancing PTEN which inhibits pAkt, and inducing (ROS-JNK-p53) loop-dependent autophagy. By modulating S1PRs, fingolimod anti-angiogenesis activity is also a help in cancer treatment. Antiproliferative effects on fingolimod at some points occur by inhibiting TRPM7, cPLA2α, SPHK1, and LPA which makes the drug an interesting object in cancer research. cPLA2α, cytosolic phospholipase A2; LPA, lysophosphatidic acid; PP2A, protein phosphatase 2A; PTEN, phosphatase and tensin homolog; S1PR, sphingosine 1-phosphate receptor; SPHK, sphingosine kinase; TRPM7, transient receptor potential cation channel, subfamily M; ROS-JNK-P53, reactive oxygen species-c-Jun N-terminal kinase-protein 53.

Since its discovery, a substantial number of studies have been conducted to explore the potential effects of fingolimod on uncontrolled cell proliferation, apoptosis, and cancer. Initially, in 1996 Suzuki et al. reported apoptotic lymphocyte reduction in rat spleen cells (Suzuki et al., 1996). Next, the Matsuda group described fingolimod-induced apoptosis in WR19L mouse lymphoma cells *in vitro* (Matsuda et al., 1998). In 2002, Permpongkosol reported antiproliferative effects of fingolimod in DU145 human prostate cancer cells (Permpongkosol et al., 2002). However, the most exciting piece of information regarding the possible antitumor effects of fingolimod came through Azuma’s publication showing the efficacy of fingolimod in inhibiting JygMC (A) breast cancer cells under both *in vitro* (2 or 10 µM) and *in vivo* (*5 mg/kg* in subcutaneous xenograft bearing nude mice) conditions. Induction of apoptosis together with reduced expression of integrins on the cancer cell surface, reduced cell adherence, and migration (metastasis) were regarded as the mechanism behind the fingolimod effect (Azuma et al., 2002). Since then, the interest and the information on the potential efficacy of fingolimod in breast cancer have been rapidly growing. Parallel with this work; fingolimod has been shown to inhibit human T cell leukemia Jurkat and B cell leukemia BALL-1 cells through dephosphorylation of Akt. Additionally, fingolimod has been shown to inhibit critical signaling pathways involved in tumor growth and metastasis, including mTOR, PIK3/Akt, and MAPK/ERK signaling pathways (Chen et al., 2018; Hou et al., 2018). Activating PP2A by fingolimod (Matsuoka et al., 2003) is another critical fingolimod effect in this context. PP2A is a tumor suppressor with decreased activity in various human cancers and a prominent target in anticancer drug development. Existing reports are suggestive of the antitumor activity of fingolimod in various experimental models of cancer, including gastric (Zheng et al., 2010), colon (Nagaoka et al., 2008), pancreatic (Shen et al., 2007), liver (Hung et al., 2008), lung (Booth et al., 2019), prostate (Pchejetski et al., 2010), ovarian (Zhang et al., 2013), and breast cancers (Rupp et al., 2021). However, it has been shown that not all of these fingolimod-induced antitumor effects are S1PR related. Indeed, activation of PP2A and the inhibition of the oncoprotein SphK1 are examples of S1PR-independent effects of fingolimod (Matsuoka et al., 2003; Vessey et al., 2007). Furthermore, analysis of human multiple myeloma samples and cell lines has revealed that 5 µM fingolimod induces ferroptosis (an iron-dependent type of cell death) and autophagy through the PP2A/AMPK pathways (Zhong et al., 2020). They also showed that fingolimod inhibits other critical regulators of ferroptosis, including Glutathione peroxidase 4 (GPX4) and soluble carrier family 7 member 11 (SLC7A11) at both the mRNA and protein levels (Zhong et al., 2020). These results are perhaps not too surprising as prior to these discoveries, it was found that S1P contributes to inflammation and cancer (Nagahashi et al., 2018). As a pleiotropic lipid mediator, S1P regulates cell survival, proliferation, migration, angiogenesis, and lymph angiogenesis together with immune cell recruitment (Nagahashi et al., 2018). The area which has attracted the most attention regarding fingolimod antitumor activity is perhaps breast cancer. S1P and another component of the S1P signaling, Sphingosine Kinase 1, are significantly upregulated in breast cancer (Nava et al., 2002; Ling et al., 2011). These recent results suggest a potential role for S1P modulators and fingolimod in the future management of breast cancer. Another potential area is the addition of fingolimod to the existing chemotherapeutic agents. Indeed, the initial experimental results here are promising and await clinical confirmation. It has also been shown that fingolimod mitigates cancer-induced bone pain and neuroinflammation in mice (Grenald et al., 2017) and alleviates bortezomib-induced neuropathic pain in rats by S1PR1 antagonism (Stockstill et al., 2018). Despite all the findings so far, the complete antitumor mechanism of fingolimod is not yet fully known and remains to be elucidated through future investigations. However, a literature review reveals that the range of concentrations and doses employed both *in vitro* and *in vivo* experiments to achieve cell or tumor growth inhibition are above and beyond the currently accepted concentrations achieved in fingolimod-treated MS patients. Therefore, it remains to be seen whether fingolimod can be effective at therapeutically acceptable doses in clinical trials in cancer patients. Figure 3 shows some of the most important targets of fingolimod in oncogenesis.

## Other S1P Receptor Modulators

Since fingolimod approval for MS therapy, other S1P receptor modulators have been developed. More S1PR selectivity (especially towards S1P1R) is of particular interest (Subei and Cohen, 2015). Currently, three more selective S1P receptor modulators, ozanimod, siponimod, and ponesimod, have received FDA approval (Shipley and Darais, 2015). These drugs have shown more selectivity for S1P receptors, shorter half-lives than fingolimod, and reduced adverse effects. Acting independently of phosphorylation of these second generation of S1PR modulators has led to faster onset of action (McGinley and Cohen, 2021; Roy et al., 2021). The success of S1PR modulators in MS and the extensive preclinical evidence in other diseases has opened a new era in pharmacology regarding the use of more selective and specific S1PR modulators. However, these newer S1PR modulators may also act other than S1PR modulation like fingolimod. Hence further investigations are warranted.

## Summary

Fingolimod has emerged as an exciting drug molecule that can be utilized in diverse pathological conditions. This phenomenon has resulted from the progressive and accelerated discoveries made on the drug’s pharmacological properties. Its close structural resemblance to sphingosine is probably a principal part of its displayed effects. Conversion in the body to fingolimod 1-phosphate allows the active metabolite to act as a potent sphingosine receptor modulator leading to immunomodulation through lymphocyte sequestration. As a functional antagonist of the S1PR1, fingolimod-P causes irreversible receptor downregulation, causing lymphocyte sequestration in lymphoid organs, leading to a marked decrease in circulating lymphocytes and endothelial barrier disruption.

With S1P being a pivotal molecule in intracellular signaling, the high-affinity binding of fingolimod to the S1PR1 leads to Gαi/o inactivation of several signaling pathways, including the PI3K/Akt/mTOR, PI3K/Rac, STAT3, STAT1, PLC, and VEGF amongst others. Fingolimod does not seem to act through the S1PR2 due to its low affinity for the receptor. It has been proposed to act as a functional antagonist of S1PR3, while effects mediated through the S1PR4 are not fully elucidated. It elicits a number of its pharmacological effects through high-affinity binding to S1PR5.

In terms of pharmacological activity, fingolimod acts as an oligodendrocyte precursor cell migration inhibitor through S1PR1 and S1PR5 regulation. Fingolimod also regulates survival, differentiation, and extension of cellular processes in oligodendrocytes. As a class 1 histone deacetylase inhibitor (HDACi), it promotes therapeutic efficacy in MS and experimental models of epilepsy and cancer, particularly in breast cancer. Clinically relevant doses of fingolimod have been shown to suppress spontaneous breast tumors development, progression, and aggressiveness in laboratory animals, and this is currently an excellent area of interest. While the rationale behind this approach is sound, it remains if it does translate to a therapeutic effect in the clinic.

Another area of interest for fingolimod is enhancing BDNF expression and anti-inflammatory effects contributing to its neuroprotective activity. While this is all well established in MS, it remains to be seen if fingolimod or any of its newer analogs can elicit similar therapeutic effects in other neurodegenerative conditions. In part from BDNF enhancement.

Various studies have firmly documented the effect of fingolimod on apoptosis. It has been proven to have the capacity to induce apoptosis in various pathological conditions, a cell-type and context-dependent property. Similarly, fingolimod enhances the autophagy markers; its effects depend on the cell context and the condition so that autophagy functions can be either pro-death or pro-survival. These fingolimod properties have been shown to account for therapeutic benefits in diverse experimental pathological conditions in laboratory animal models. On this basis, there are currently several ongoing clinical trials evaluating fingolimod in patients with MS disease or stroke.

Protein phosphatase 2 (PP2A) is another well-established target of fingolimod. By activating PP2A, fingolimod plays a principal role as a regulator of cell cycle/division and growth, apoptosis, and an inhibitor of various signal transduction pathways relevant to inflammation. Coupled with its other anti-inflammatory properties, such as inhibition of phospholipase C, it theoretically provides the rationale for its evaluation as an anti-inflammatory agent.

Furthermore, novel fingolimod derivatives have been synthesized and are currently being evaluated for safety and selective efficacy in various diseases. It, therefore, remains to be seen if fingolimod and or its novel derivatives may gain approval in other pathological conditions and in particular in other neurodegenerative conditions.
